# An evaluation of TAZ and YAP crosstalk with TGFβ signalling in canine osteosarcoma suggests involvement of hippo signalling in disease progression

**DOI:** 10.1186/s12917-018-1651-5

**Published:** 2018-11-26

**Authors:** Anita K. Luu, Courtney R. Schott, Robert Jones, Andrew C. Poon, Brandon Golding, Roa’a Hamed, Benjamin Deheshi, Anthony Mutsaers, Geoffrey A. Wood, Alicia M. Viloria-Petit

**Affiliations:** 1 Department of Biomedical Sciences, Ontario Veterinary College, University of Guelph, 50 Stone Road East, Guelph, ON N1G 2W1 Canada; 2 Department of Pathobiology, Ontario Veterinary College, University of Guelph, 50 Stone Road East, Guelph, ON N1G 2W1 Canada; 3 Medical City Forth Worth, HCA affiliated Hospital, 900 8th Ave, Fort Worth, TX 76104 USA

**Keywords:** Canine osteosarcoma, Hippo signalling, TGFβ, YAP1, TAZ, WWTR1, Prognostic marker, Metastasis

## Abstract

**Background:**

Osteosarcoma (OSA) is the most common bone cancer in canines. Both transforming growth factor beta (TGFβ) and Hippo pathway mediators have important roles in bone development, stemness, and cancer progression. The role of Hippo signalling effectors TAZ and YAP has never been addressed in canine OSA. Further, the cooperative role of TGFβ and Hippo signalling has yet to be explored in osteosarcoma. To address these gaps, this study investigated the prognostic value of TAZ and YAP alone and in combination with pSmad2 (a marker of active TGFβ signalling), as well as the involvement of a TGFβ-Hippo signalling crosstalk in tumourigenic properties of OSA cells in vitro. An in-house trial tissue microarray (TMA) which contained 16 canine appendicular OSA cases undergoing standard care and accompanying follow-up was used to explore the prognostic role of TAZ, YAP and pSmad2. Published datasets were used to test associations between *TAZ* and *YAP* mRNA levels, metastasis, and disease recurrence. Small interfering RNAs specific to TAZ and YAP were utilized in vitro alone or in combination with TGFβ treatment to determine their role in OSA viability, proliferation and migration.

**Results:**

Patients with low levels of both YAP and pSmad2 when evaluated in combination had a significantly longer time to metastasis (log-rank test, *p* = 0.0058) and a longer overall survival (log rank test, *p* = 0.0002). No similar associations were found for TAZ and YAP mRNA levels. In vitro, TAZ knockdown significantly decreased cell viability, proliferation, and migration in metastatic cell lines, while YAP knockdown significantly decreased viability in three cell lines, and migration in two cell lines, derived from either primary tumours or their metastases. The impact of TGFβ signaling activation on these effects was cell line-dependent.

**Conclusions:**

YAP and pSmad2 have potential prognostic value in canine appendicular osteosarcoma. Inhibiting YAP and TAZ function could lead to a decrease in viability, proliferation, and migratory capacity of canine OSA cells. Assessment of YAP and pSmad2 in larger patient cohorts in future studies are needed to further elucidate the role of TGFβ-Hippo signalling crosstalk in canine OSA progression.

**Electronic supplementary material:**

The online version of this article (10.1186/s12917-018-1651-5) contains supplementary material, which is available to authorized users.

## Background

Osteosarcoma (OSA) is the most commonly diagnosed primary cancer of the bone in both humans and dogs, but it is about fourteen times more common in dogs, with an estimated incidence of 13.9/100,000 [1]. Most canine OSAs develop in large and giant dog breeds, are appendicular in location, and tend to develop pulmonary metastasis [1]. The standard of care (SOC) for canine osteosarcoma (OSA) currently consists of limb amputation or limb-sparing surgery and chemotherapy. With this treatment, the median survival time is 8–12 months and metastasis to the lungs is primarily responsible for patient’s mortality [2, 3]. As such, there has been an increased focus on discovering novel prognostic markers and molecular targets to improve patient outcome.

Transforming growth factor beta (TGFβ) exists in three different isoforms (TGFβ 1, 2 and 3). All of these, in addition to other members of the TGFβ superfamily of secreted proteins (in particular bone morphogenetic proteins/BMPs), have been implicated in bone formation, remodeling and bone metastasis [4, 5]. The three TGFβ isoforms classically signal via the Smad pathway, involving Smad2/3 and Smad4. This is initiated by TGFβ binding to a TGFβ receptor type II (TβRII) homodimer, which facilitates the formation of a complex with a TGFβ receptor type I (TβRI) homodimer. In this tetrameric complex, TβRII (a constitutively active kinase) phosphorylates and activates TβRI, leading to the recruitment of the receptor-activated Smads (R-Smads), Smad2 and Smad3. Smad 2/3 recruitment to the TβR complex leads to their c-terminal phosphorylation and subsequent activation by TβRI, which enables them to form a complex with the co-Smad, Smad4. The R-Smads and co-Smad complex then translocates to the nucleus to modulate gene expression through cooperation with other transcription factors, co-activators, and co-repressors (reviewed in [6]).

In respect to OSA, several studies support an important role of TGFβ in the invasive/aggressive behavior of both human and canine OSA. Human OSA patients with high levels of TGFβ3 in tumour tissue have a shorter disease-free survival, whereas human patients with high grade OSA had significantly greater tumour expression of TGFβ1 when compared to low-grade OSA patients [7, 8]. In addition, TGFβ signalling promotes growth, migration, and invasiveness in both human and canine OSA cell lines [9–11], and TGFβ1 induces de-differentiation of OSA cells into self-renewing cancer stem cells [12]. Given the demonstrated roles of cancer stem cells in resistance to various therapy modalities, such as conventional chemotherapy, radiotherapy, and molecular targeted therapy, these latter findings suggest that high TGFβ signalling in OSA, apart from promoting metastasis, could additionally contribute to patient mortality by driving therapy resistance [13].

The transcriptional modulator, referred to as WW domain-containing transcription regulator 1 (WWTR1), also known as transcriptional co-activator with a PDZ binding motif (TAZ, the acronym we will use from now on), has been shown to be important for regulating Smad (the downstream mediator of TGFβ signalling) transcriptional activity [14]. Both TAZ and its paralogue YAP1 (Yes-associated protein 1) act as co-activators for a number of transcription factors, and were first known for their role in the Hippo pathway [15]. However, later studies indicated that they also facilitate nuclear sequestration of Smads and subsequent transcriptional activity [14, 15]. This crosstalk between TGFβ and Hippo signalling might be particularly important in osteosarcoma biology. TAZ mediates mesenchymal stem cell differentiation into osteoblasts via its association with Runx2 [16, 17], as well as osteogenic differentiation of bone marrow stromal cells downstream of TGFβ [18]. Further, TAZ is required to maintain self-renewal of embryonic stem cells [14, 19], and was shown to confer invasive properties, self-renewal capacity and chemoresistance to cancer cells [20, 21]. Recent findings indicate that TAZ mediates TGFβ-induced carcinoma progression, through the promotion of metastasis and the cancer stem cell phenotype [22].

The role of Hippo signalling in sarcomas is also well documented. TAZ and YAP were both found to be commonly activated in human sarcomas, with 2/3 of sarcomas harboring nuclear TAZ and 1/2 harboring nuclear YAP [23]. In this study, high levels of *TAZ* mRNA were found to be associated with reduced overall survival in dedifferentiated liposarcoma [23]. With regard to OSA, high TAZ/YAP expression in tumour tissue samples was found to correlate with poor overall survival in human OSA [24], and an in vitro study showed that YAP promotes chemoresistance in human OSA cell lines [25]. Treatment of human OSA cells with chemotherapeutics doxorubicin and methotrexate was shown to cause degradation of MST1/2 and decreases in LATS1/2 protein levels, the upstream regulators of TAZ/YAP. This subsequently caused an increase in nuclear YAP levels, promoting cell proliferation and chemoresistance [25]. The nuclear localization of Hippo mediators is important for their ability to interact with TEAD (TEA domain DNA-binding family of transcription factors) and activate downstream gene targets to promote proliferation, survival and invasiveness [25].

In veterinary oncology and to the best of our knowledge, TAZ has only been explored in canine mammary tumours, where it was observed that high grade (grade III) tumours had high nuclear expression of TAZ [26]. In vitro, canine mammary tumours strongly express TAZ and disruption of TAZ/YAP-TEAD with verteporfin treatment induces cell apoptosis and reduces migratory and invasive properties [27].

Thus, based on the aforementioned evidence, we hypothesized that levels of nuclear phosphorylated Smad2 (pSmad2, indicative of activated TGFβ signalling), TAZ, YAP or combinations of these markers, will associate with established markers of poor prognosis, metastatic disease and overall patient survival in canine OSA. Furthermore, TAZ and YAP depletion will decrease cell migration and proliferation in canine OSA cell lines. To address these hypotheses, this study employed a pilot tissue microarray (TMA) containing 41 OSA tumour samples, 16 of which were derived from patients with appendicular OSA that were treated with the SOC and had accompanying follow-up. We also investigated the TGFβ-TAZ/YAP relationship in vitro, using siRNA specific to TAZ and YAP in combination with TGFβ treatment to determine its role in promoting tumourigenic properties. Results show that low levels of YAP and pSmad2 combined associate with longer time to metastasis and longer overall survival, while both TAZ and YAP depletion, and TGFβ signalling activation, impacted cell viability, proliferation and migration of OSA cell lines in a cell line-dependent manner.

## Results

### Clinical data

A total of sixteen appendicular canine OSA patients that underwent SOC were considered in patient analyses. Specifically, the SOC consisted of limb amputation or limb-sparing surgery and 1 to 6 cycles of carboplatin (depending on the patients), which was administered every 3 weeks at a dose of 300 mg/m^2^ IV, starting 10–14 days post surgery. The patient data set had a larger representation of male (75%) as compared to female (25%) patients. The average age and weight of patients plus standard deviation, were 8.01 ± 1.73 years and 37.1 ± 10.3 kg, respectively at the time of diagnosis. Alkaline phosphatase (ALP) status and classification of “high”, “low” or “normal” was determined by serum biochemistry test at the time of diagnosis; 25% of patients had high ALP. Of all 16 patients with follow-up, 62.5% were classified as high for pSmad2 levels, while 37.5% were classified as low for pSmad2 levels. This distribution was similarly observed for patients when considering YAP levels: 64% and 36% of patients were classified as having high and low YAP levels, respectively. In terms of TAZ levels, 56.3% of patients were classified as having high TAZ levels and 44.7% were classified as having low TAZ levels. All patient data and their corresponding classification for ALP, pSmad2, TAZ, and YAP expression are indicated in Table 1. Histologic grade classification by the two existing systems for canine OSA is also included.

**Table 1:** Clinical and histopathological characteristics of appendicular OSA cases included in metastasis and survival analyses

Case	BREED	SEX	Age Dx (Y)	Weight Dx (kg)	Location	ALP Status	Survival Days	Metastasis	pSmad2 Levels	TAZ Levels	YAP Levels	Grade	
												Kirp	Louko
1	Standard Poodle	CM	10.55	23.6	right proximal tibia	N	91	84	High	High	N/A	3	3
2	Doberman	SF	5.99	32.4	right distal femur	H	116	93	High	High	Low	2	3
3	Rottweiler	M	6.30	53.0	left proximal humerus	H	130	N/A	High	Low	Low	2	1
4	Australian Shepherd	CM	8.32	24.6	right proximal humerus	N	148	78	High	High	High	2	2
5	Rottweiler	CM	8.26	50.0	right distal radius	H	165	131	High	Low	High	2	2
6	Greyhound	SF	8.87	26.0	right distal femur	N	215	N/A	High	Low	High	3	2
7	Mixed breed	CM	8.30	37.4	left distal radius	N	218	167	Low	High	High	2	1
8	Rottweiler	CM	4.66	53.4	left proximal humerus	N	282	182	Low	High	High	2	3
9	Golden Retriever	CM	10.99	32.2	left proximal tibia	N	294^a^	247	Low	Low	High	2	1
10	Mixed breed	CM	10.02	42.0	left distal tibia	N	301	N/A	High	High	High	2	1
11	Mixed breed	CM	7.25	51.0	left distal tibia	N	381	N/A	High	High	High	2	2
12	Doberman	SF	5.91	41.0	right distal radius	N	485^a^	485^a^	High	Low	High	3	3
13	Mixed breed	CM	8.03	28.4	left proximal femur	N	605	604	Low	Low	Low	2	1
14	Greyhound	CM	9.02	29.4	right distal femur	N	1005	1005^a^	High	High	N/A	2	1
15	Mixed breed	CM	8.25	37.2	left proximal femur	N	1386	1386^a^	Low	High	Low	2	1
16	Greyhound	SF	7.43	32.4	left distal tibia	H	1091^a^	1091^a^	Low	Low	Low	2	1

*MC* male castrated, *FS* female spayed, *ALP* alkaline phosphatase status, *H* high, *N* normal, *L* low; Kirp, Grade assigned using criteria as explained in Kirpensteijn et al. [28]; Louko, Grade assigned using criteria as explained in Loukopoulos et al. [29];

*N/A* Data not available

^a^Data was censored for Kaplan-Meier analysis

### pSmad2, TAZ and YAP Immunolabelling

**Fig. 1:**
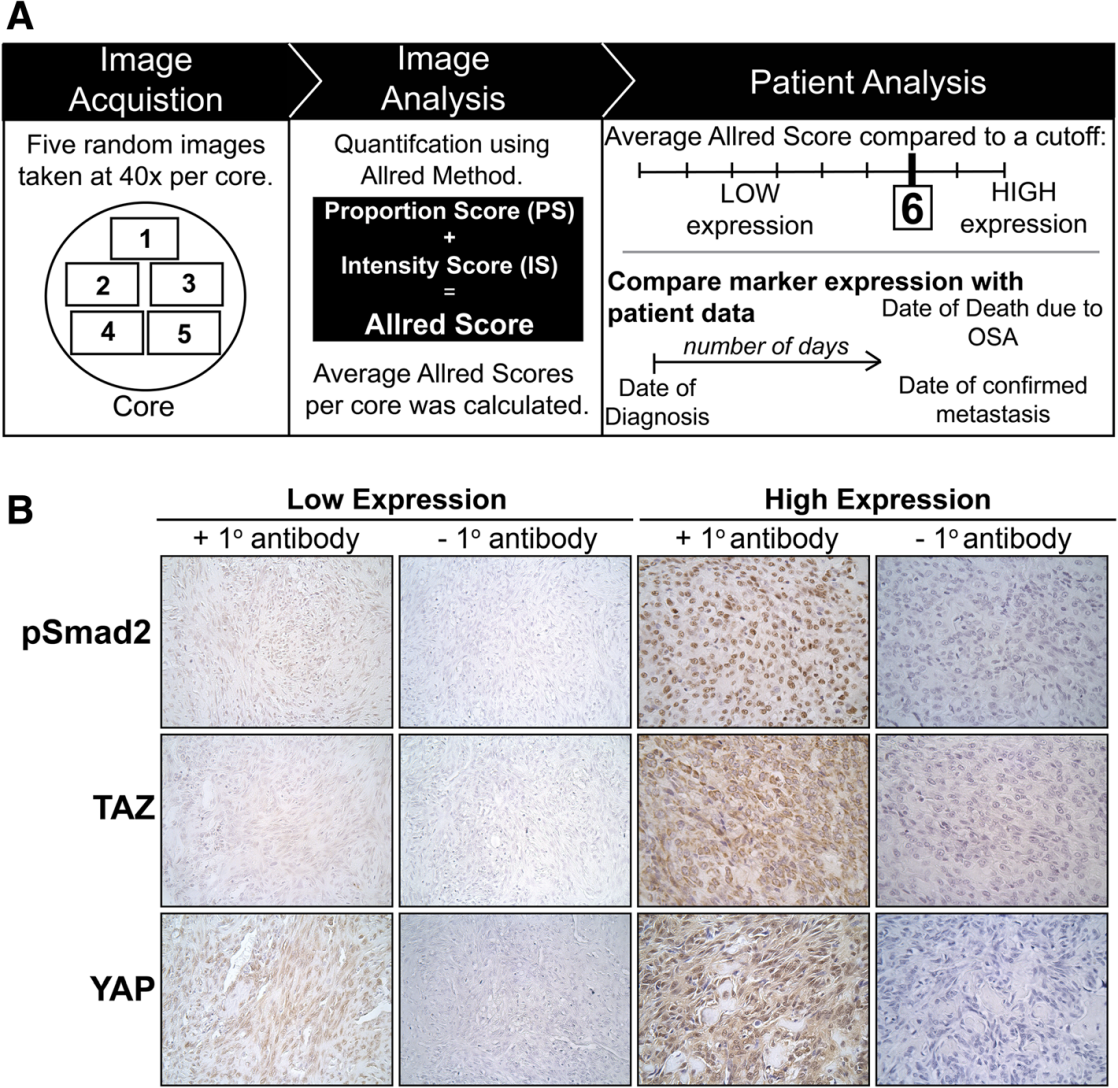
Workflow of TMA quantification and pSmad2, TAZ and YAP in canine OSA immunolabelling. Workflow for TMA image acquisition, analysis and patient analysis described in (**a**). Representative images of low and high pSmad2, TAZ and YAP immunolabeling (+ 1° antibody) and respective negative controls (- 1° antibody) in canine OSA tumour tissue at 40X (**b**)

To determine antibody specificity, immunoblotting was performed on canine OSA cell lines. The immunoblot for YAP demonstrates a prominent band at the expected position of 65 kDa, with possible reactivity with TAZ. Similarly for TAZ, the immunoblot shows a prominent band at the expected position (55 kDa), with some possible reactivity with YAP and other possible lower and higher molecular weight forms of TAZ (Additional file 1: Figure S1); pSmad2 showed only one band at the expected molecular weight (60 KDa), which was significantly enhanced by TGFβ1 treatment, as expected (data not shown). In tumour tissue, pSmad2 immunolabelling was predominantly nuclear, while TAZ and YAP were found to be both cytoplasmic and nuclear. As expected, the intensity and level of staining for the markers of interest were variable amongst tumour cores from different cases. Representative images for high and low for pSmad2, TAZ and YAP expression, and their respective negative control (no primary antibody) are shown in Fig. 1b.

### No associations between pSmad2, TAZ, or YAP levels, and patient characteristics, histologic grade or alkaline phosphatase status

Using a chi square test, no significant associations were found between pSmad2, TAZ, and YAP levels and breed, or sex (Table 2). When considering histologic grade or ALP status at the time of diagnosis, no significant associations were found between pSmad2 levels, TAZ or YAP, and either the Kirpensteijn et al. [28] or the Loukopoulos et al [29] grading system, or the ALP status (Table 3).

**Table 2:** Associations between pSmad2, TAZ and YAP levels and patient sex and breed

Patient Characteristic	pSmad2		*p* value	TAZ		*p* value	YAP		*p* value
	High	Low		High	Low		High	Low	
Sex									
Castrated Male	6	5	0.5589	8	3	0.1296	7	2	0.2382
Male (Intact)	1	0		0	1		0	1	
Spayed Female	3	1		1	3		2	2	
Breed			0.2101			0.1967			0.8030
Non-Mixed	8	3		5	6		6	3	
Mixed	2	3		4	1		3	2

**Table 3:** Associations between pSmad2, TAZ and YAP levels and established prognostic factors

Histopathological Characteristics	pSmad2		*p* value	TAZ		*p* value	YAP		*p* value
	High	Low		High	Low		High	Low	
Tumour Grade									
Kirpensteijn et al. (2002) [28]									
1	0	0	0.1366	0	0	0.3747	0	0	0.2549
2	7	6		8	5		7	5	
3	3	0		1	2		2	0	
Loukopoulos et al. (2007) [29]			0.0907			0.6832			0.1629
1	3	5		4	4		3	4	
2	4	0		2	2		4	0	
3	3	1		3	1		2	1	
Serum ALP^a^ Status			0.5510			0.1457			0.0524
High	3	1		1	3		8	2	
Normal	7	5		8	4		1	3	
Low	0	0		0	0		0	0	

^a^ALP: Alkaline phosphatase

### Correlations of pSmad2, TAZ and YAP levels and metastasis and overall survival

**Fig. 2:**
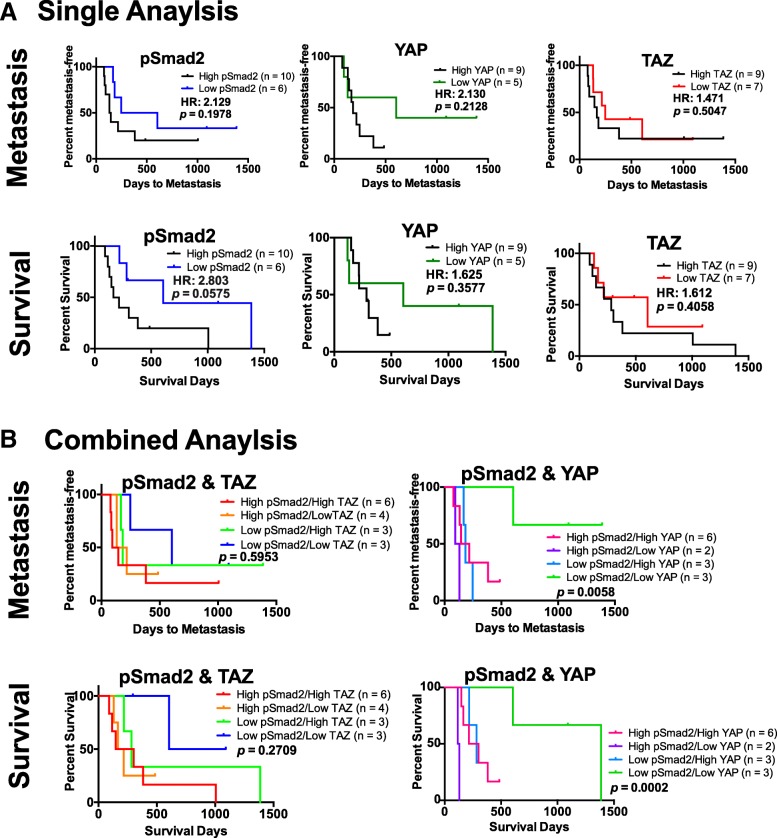
Kaplan-Meier plots depicting the correlation pSmad2, TAZ and YAP levels and time to metastasis and overall survival. No significant difference was found between the number of days to metastasis and overall survival when considering pSmad2, TAZ and YAP alone (**a**) or pSmad2 and TAZ in combination (**b**, left). Significant difference in time to metastasis and overall survival was observed when considering pSmad2 and YAP in combination (**b**, right). All statistical analyses were performed using the log-rank (Mantel-Cox) test

No significant differences were found in the time to metastasis and overall survival for patients that expressed high levels of pSmad2, TAZ or YAP compared to those that expressed low levels of these markers when they were evaluated alone (Fig. 2a). Interestingly, when pSmad2 and YAP were evaluated in combination and reclassified into four groups (Fig. 2b), there was a significant difference observed amongst the groups in the time to metastasis (log-rank/Mantel-Cox test, *p* = 0.0058) and overall survival (log rank/Mantel-Cox test, *p* = 0.0002). These trends were not observed when pSmad2 and TAZ were evaluated in combination for time to metastasis nor overall survival. The results obtained suggest that canine patients with low levels of both pSmad2 and YAP have a later time to metastasis and a longer overall survival.

### No association between *TAZ/WWTR1* or *YAP* mRNA levels and metastasis development, recurrence, and overall survival in human and canine OSA

**Fig. 3:**
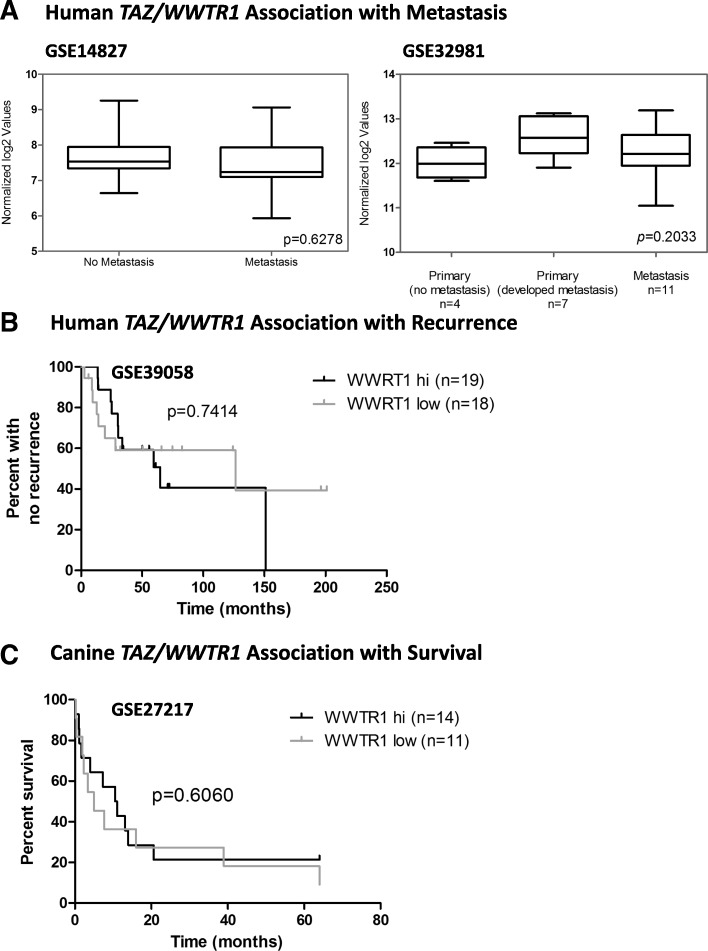
Associations between TAZ mRNA levels and metastasis, recurrence or OS. No significant differences were observed in TAZ mRNA levels in human patients that did not develop metastases, compared to patients that did metastasize (**a**, left panel). Similarly, no statistical significance was achieved when comparing TAZ mRNA levels in non-metastatic primary tumours versus primary tumours that developed metastasis, or between primary tumours and metastases (**a**, right panel). No significant differences were observed in recurrence for human patients with high versus low TAZ mRNA (**b**), or for OS in canine patients with hi versus low TAZ mRNA (**c**)

**Fig. 4:**
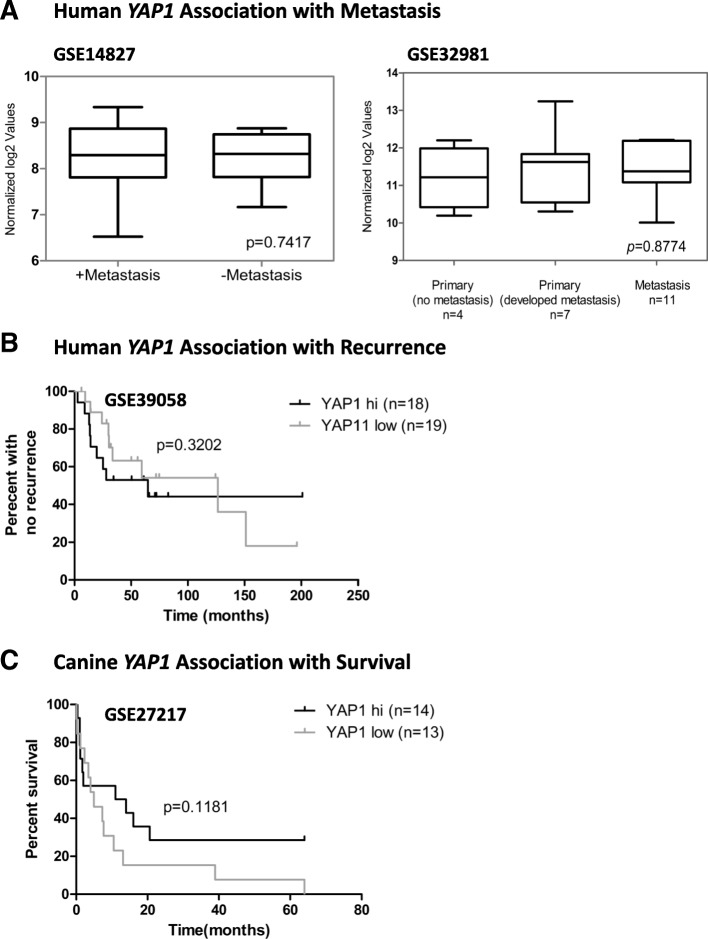
Associations between YAP1 mRNA levels and metastasis, recurrence or OS. No significant differences were observed in YAP mRNA levels in human patients that did not develop metastases, compared to patients that did metastasize (**a**, left panel). Similarly, no statistical significance was achieved when comparing YAP mRNA levels in non-metastatic primary tumours versus primary tumours that developed metastasis, or between primary tumours and metastases (**a**, right panel). No significant differences were observed in recurrence for human patients with high versus low YAP mRNA (**b**), or for OS in canine patients with hi versus low YAP mRNA (**c**)

To investigate whether the associations suggested by our analysis of TAZ and YAP proteins in canine OSA could also be observed in human OSA, we compared TAZ and YAP mRNA levels among different groups of patients using datasets from previously published studies that were deposited in the Gene Expression Omnibus (GEO) public database. We did not observe significant differences in TAZ or YAP mRNA levels in primary tumours derived from patients that develop versus patients that did not developed metastasis after tumour resection in two independent datasets, Kobayashi et al. 2010 (accession # GSE14827 [30]) and Namlos et al. 2012, (accession # GSE32981 [31]) (Figs. 3a and 4a, left and right plots, respectively). Similarly, we did not observe significant differences in TAZ or YAP mRNA levels in primary tumour tissue as compared to tissue from actual metastases in one data set Namlos et al. 2012, (accession # GSE32981 [31]) (Figs. 3a and 4a, respectively, right plot), or between tumours derived from patients that did not recur after treatment as compared to patients that recurred (Kelly et al. 2013, accession # GSE39058 [32]) (Figs. 3b and 4b, respectively). Analysis of one available dataset for canine OSA showed no association between TAZ or YAP mRNA levels and overall survival (Scott et al. 2011, accession # GSE27217 [33]) (Figs. 3c and 4c, respectively). Similarly, no associations with metastasis, recurrence, or OS were found when we used the same data sets to look for correlations between the aforementioned parameters and TAZ or YAP mRNA levels in combination with the mRNA level of genes in a TGFβ signature [34] comprised of *TGFB1* itself plus the following: *COL1A1*, *COL6A1*, *COL6A3*, *MMP11*, *MMP14* and *POSTN* (data not shown). To further explore whether the associations observed in the TMA analyses reflect a role for TAZ and/or YAP in canine OSA and whether or not their function is influenced by canonical TGFβ signaling activation, in vitro studies were completed.

### Characterization of TGFβ signalling and hippo pathway mediators in canine osteosarcoma cell lines

**Fig. 5:**
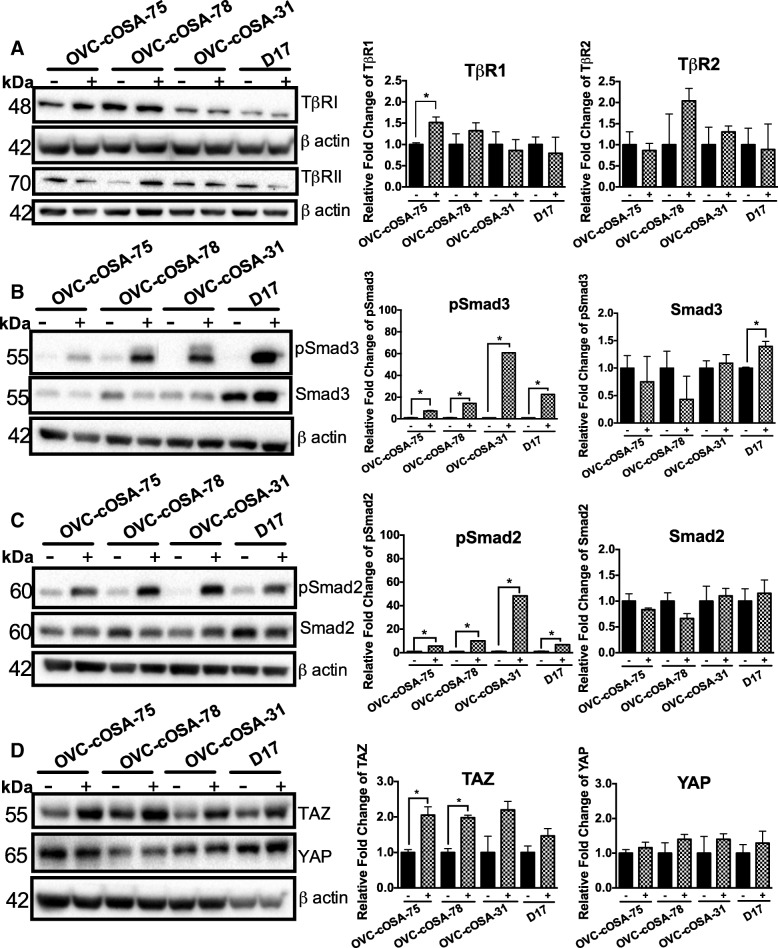
All canine OSA cell lines express TGFβRI, TGFβRII, respond to TGFβ1 treatment and have basal levels of Hippo mediators TAZ and YAP. Cells were serum starved for 6 h and either stimulated with 5 ng/mL TGFβ1 (+) or not (−) for 24 h. Representative immunoblots and densitometry analysis showing the levels of TGFβRI and TGFβRII (**a**), pSmad3 and Smad 3 (**b**), pSmad2 and Smad2 (**c**) and TAZ and YAP (**d**), *n* = 3. Phospho-protein levels were normalized to native protein levels, and all native protein levels were normalized to β actin (loading control) and compared to the control (−) to determine relative differences. Independent t-test was used to determine significant differences between control and treated groups, * indicates *p* < 0.05, ** indicates *p* < 0.010, error bars depict average ± SEM

To first determine the presence of both TGFβ receptors, as well as the degree of Smad activation in response to exogenous TGFβ and its effects on Hippo pathway mediators, TAZ and YAP, immunoblotting analyses was completed on four canine OSA cell lines, two derived from primary tumours (OVC-cOSA-75 and OVC-cOSA-78), and two derived from metastatic colonies (OVC-cOSA-31 and D17). As expected, all cell lines expressed both TβRI and TβRII. The primary cell lines appeared to have higher levels of TβRI when compared to the metastatic cell lines, while there were no obvious patterns when considering TβRII levels (Fig. 5a). To determine the extent of canonical TGFβ signalling activation, the levels of phosphorylated (active) Smad2 and Smad3 were determined after 5 ng/mL of TGFβ treatment. All cell lines robustly responded to exogenous TGFβ1 as demonstrated by the large increase in phosphorylated Smad3 and Smad2 (pSmad3 and pSmad2, shown in Fig. 5b and c, respectively). TAZ levels increased in response to TGFβ1 treatment (significantly so for OVC-cOSA-75 and OVC-cOSA-78 cell lines only), while YAP levels remained fairly similar between treatment groups (Fig. 5d).

### Cell-line dependent effects of TAZ and YAP depletion on cell viability

**Fig. 6:**
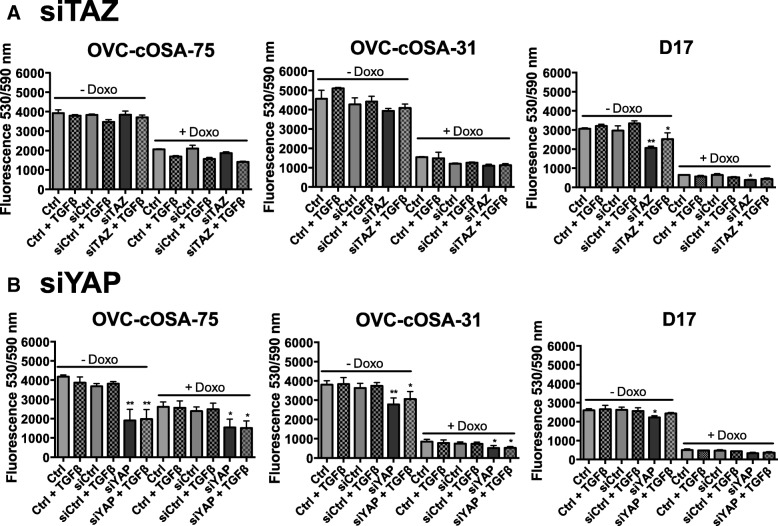
Effect of TAZ or YAP knockdown plus and minus TGFβ on cell viability of canine OSA cell lines. Graphs depict the fluorescence readings at 530/590 nm when TAZ (**a**) or YAP (**b**) was depleted in OVC-cOSA-75, OVC-cOSA-31, and D17 cells, which were stimulated or not with 5 ng/mL TGFβ in the presence (+ Doxo) or absence (−Doxo) of Doxorubicin. Readings were first blank-corrected to fluorescence values obtained for the media only control. Bars depict the average ± SEM from two independent experiments plated in duplicate. Asterisks depict statistical differences as determined by a one-way ANOVA (Kruskal-Wallis test) with Dunn-Sidak correction to identify significant differences relative to control within each, the –Doxo and + Doxo treatment groups; * indicates *p* < 0.05, ** indicates *p* < 0.010. TAZ knockdown decreased cell viability in the D17 cell line both in the presence and absence of Doxorubicin. YAP knockdown decreased cell viability in the presence (OVC-cOSA-75, OVC-cOSA-31) and absence (all three cell lines) of Doxorubicin

To determine the role of Hippo pathway mediators and TGFβ signaling on OSA cell viability, TAZ and YAP were depleted in three cell lines using siRNA (siTAZ and siYAP), and the cells were subsequently treated or not with TGFβ, followed by exposure (or not) to Doxorubicin, a drug commonly used for SOC of canine osteosarcoma [2], and one that in our hands has been reliable for in vitro experimentation. TAZ knockdown significantly reduced viability of D17 cells relative to control (Ctrl) both in the absence and presence of Doxorubicin, and both in the presence or absence of TGFβ for cells that were not treated with Doxorubicin, but has no effect on the other two cell lines (Fig. 6a). In contrast, YAP knockdown significantly reduced cell viability in all three cell lines relative to control (Ctrl), and this occurred both in the presence or absence of doxorubicin, and in the presence or absence of TGFβ, for OVC-cOSA-75 and OVC-cOSA-31, but not for D17, where the effect was only significant in the absence of doxorubicin and in the absence of TGFβ (Fig. 6b). Taken together, these results suggest that YAP is a key mediator of canine OSA cell viability and can influence their response to chemotherapy, while TAZ role is more limited.

### Cell-line dependent effects of TAZ and YAP depletion on cell proliferation

**Fig. 7:**
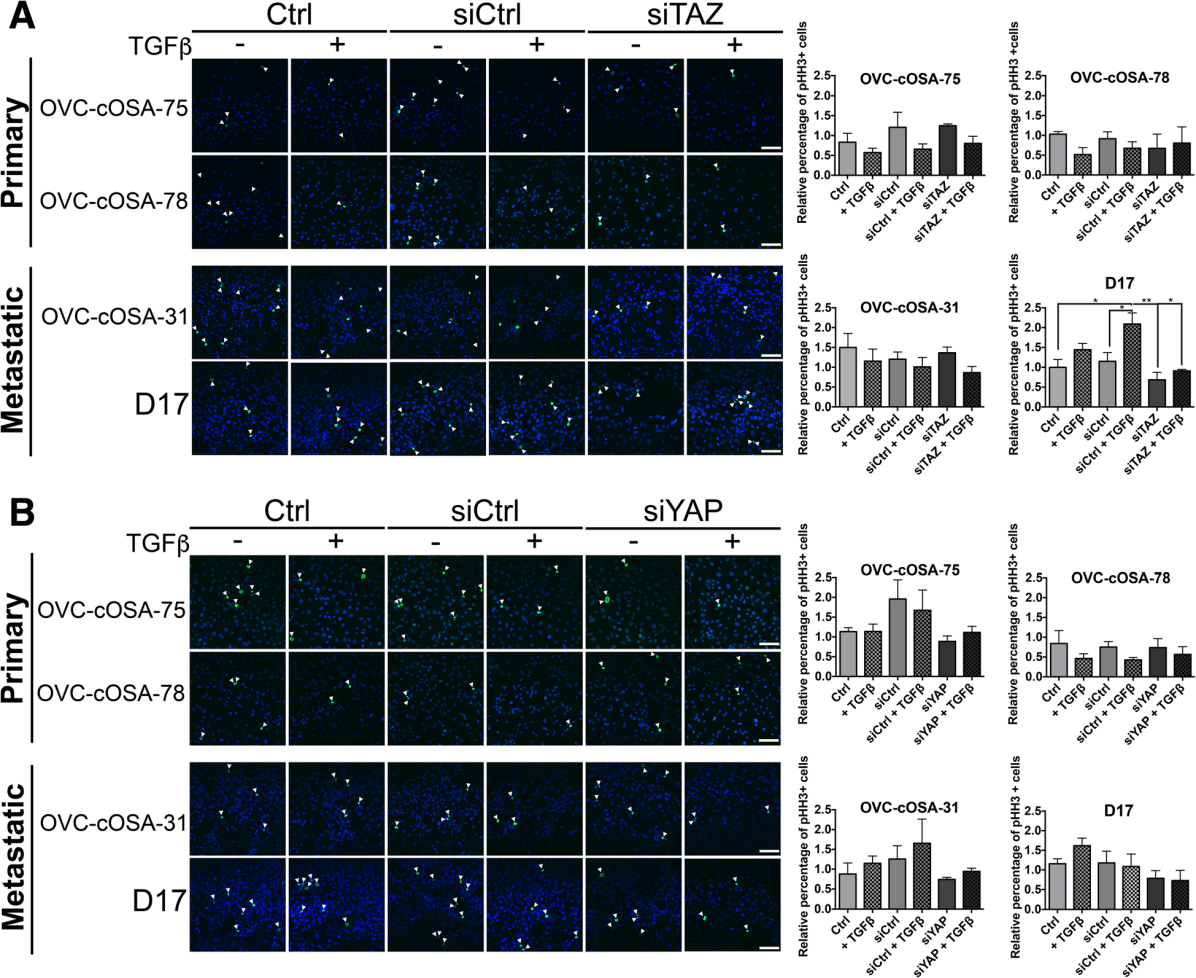
Effect of TAZ or YAP knockdown plus and minus TGFβ on cell proliferation of canine OSA cell lines. Representative immunofluorescence images of the mitotic marker phosphor-histone H3 (pHH3) taken at 20X objective lens for primary tumour-derived cell lines (**a**), and metastatic cell lines (**b**). Graphs depict the relative percentage of pHH3 positivity cells, as determined by dividing the number of pHH3 cells by the number of cells in the field of view. Five images were taken per experimental group and averaged. Error bars depict average ± SEM from three independent experiments. Scale bar = 100 μm. Asterisks depict statistical differences as determined by a two-way ANOVA with post-hoc Tukey-Kramer, * indicates *p* < 0.05, ** indicates *p* < 0.010, *** indicates *p* < 0.0010

Since a reduction in cell viability caused by YAP or TAZ knockdown in various OSA cell lines could result from an effect on cell survival, cell proliferation, or both, we next focused on assessing the role of TAZ, YAP, and TGFβ signalling on cell proliferation exclusively. For this purpose, we depleted TAZ and YAP with siRNA (siTAZ and siYAP) and subsequently cultured the cells in the presence or absence of TGFβ1 under serum starvation conditions for optimal canonical signaling activation (the effectiveness of siRNAs at downregulating TAZ and YAP expression can be seen in Additional file 2: Figure S2 and Additional file 3: Figure S3). The mitotic marker phospho-histone H3 (pHH3) was used to fluorescently label proliferating cells (Fig. 7). All cell lines demonstrated a low basal proliferating rate (~ 1–2%) under serum starvation conditions as seen in the untreated control (Ctrl) and siRNA control (siCtrl) groups. TAZ depletion decreased the relative percentage of pHH3 positive cells in the D17 cell line, while having no significant effect on the OVC-cOSA-75, OVC-cOSA-78, and OVC-cOSA-31 cell lines. Also in the D17 cell line, TGFβ treatment significantly increased pHH3 positivity, better visualized when comparing the siCtrl and siCtrl + TGFβ groups (*p =* 0.0479). However, when TAZ was depleted alone and in combination with TGFβ treatment, there was a reduction in pHH3 positivity, which became significant when comparing siCtrl + TGFβ to siTAZ (*p* = 0.0029), and to siTAZ + TGFβ groups (*p* = 0.011). This was not observed for OVC-cOSA-75, OVC-cOSA-78 and OVC-cOSA-31 (Fig. 7a). In terms of YAP, there was no statistically significant change observed in pHH3 positivity upon knockdown in any of the cell lines (Fig. 7b), although a slight decrease in pHH3 positivity was seen in OVC-cOSA-31 and D17 cells relative to Ctrl and siCtrl, and this was both in the absence or presence of TGFβ treatment (Fig. 7b). These results suggest that TAZ (and possibly YAP), mediate cell proliferation in OSA cell lines in a cell line-dependent manner and in both the presence and absence of activated TGFβ signalling.

### TAZ and TGFβ signaling crosstalk in D17 cells

**Fig. 8:**
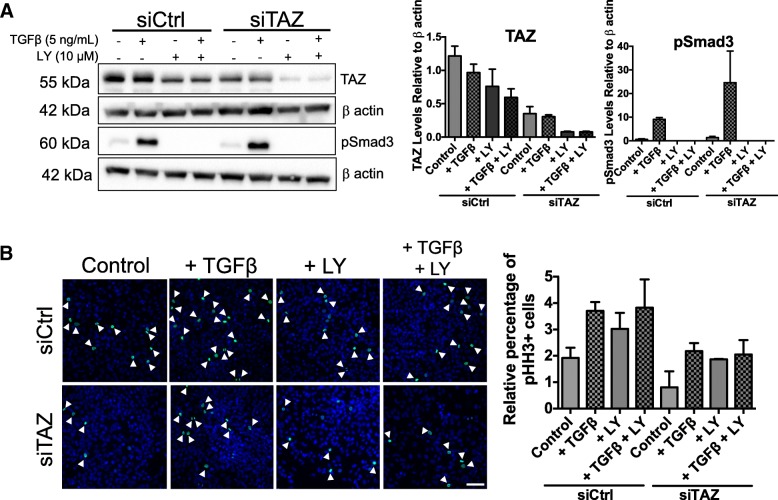
Effect of TGFβ receptor I inhibition by LY2157299 on TAZ levels and cell proliferation in D17 cells. Representative immuoblots and densitometry analysis of TAZ and pSmad3 protein levels when TAZ levels were depleted (siTAZ) or not (siCtrl), and cells were treated with nothing (Control), 5 ng/mL TGFβ (+ TGFβ), 10 μM of LY2152799 (+ LY) or combination treatment (+ TGFβ + LY). Experimental groups were normalized to loading control β-actin. Graphs depict the average fold change in TAZ or pSmad3 expression relative to siCtrl non-treated group ± SEM from two independent experiments (**a**). LY inhibited Smad3 phosphorylation and decreased TAZ levels compared to non-LY treated cells, suggesting that suppressing active TGFβ signaling could decrease TAZ levels in D17 cells. **b** Representative immunofluorescence images of the mitotic marker phospho-histone H3 (pHH3) taken at 20X objective lens and graph depicting the relative percentage of pHH3 positive cells; bars depict average ± SEM from three independent experiments. Scale bar = 100 μm

The above described effects of TAZ knockdown on D17 cells, where reducing TAZ levels not only inhibited proliferation in the absence of TGFβ, but also blocked the growth-stimulatory effects of this cytokine, were suggestive of a crosstalk between TGFβ signaling and TAZ, whereby low levels of TAZ can negatively modulate pro-tumorigenic properties of TGFβ in this cell line. To explore this possibility, we conducted the same experiment in the presence and absence of a specific inhibitor of the TGFβ receptor type I (referred to here as LY), which blocks the activation of canonical (Smad) TGFβ signaling. LY treatment reduced the levels of TAZ in D17 cells (Fig. 8a), suggesting that basal TAZ expression in D17 cells is dependent on TGFβ signaling, possibly resulting from the capacity of D17 cells to secrete its own TGFβ (autocrine signalling), as these experiments were performed under serum starvation conditions. Although the results were not statistically significant due to high variability among the replicates, the average percentage of proliferating cells under TGFβ stimulation was equally reduced by TAZ knockdown independently of whether or not TGFβ signaling was blocked (compare siCtrl + TGFβ versus siTAZ + TGFβ and versus siTAZ + TGFβ + LY in Fig. 8b). These results support the existence of a crosstalk between TGFβ signalling and TAZ in D17 cells, whereby TAZ expression is modulated by TGFβ. However, TGFβ signaling inhibition does not significantly enhance the effect of TAZ knockdown in cell proliferation.

### Cell line dependent effects of TAZ and YAP in cell migration

**Fig. 9:**
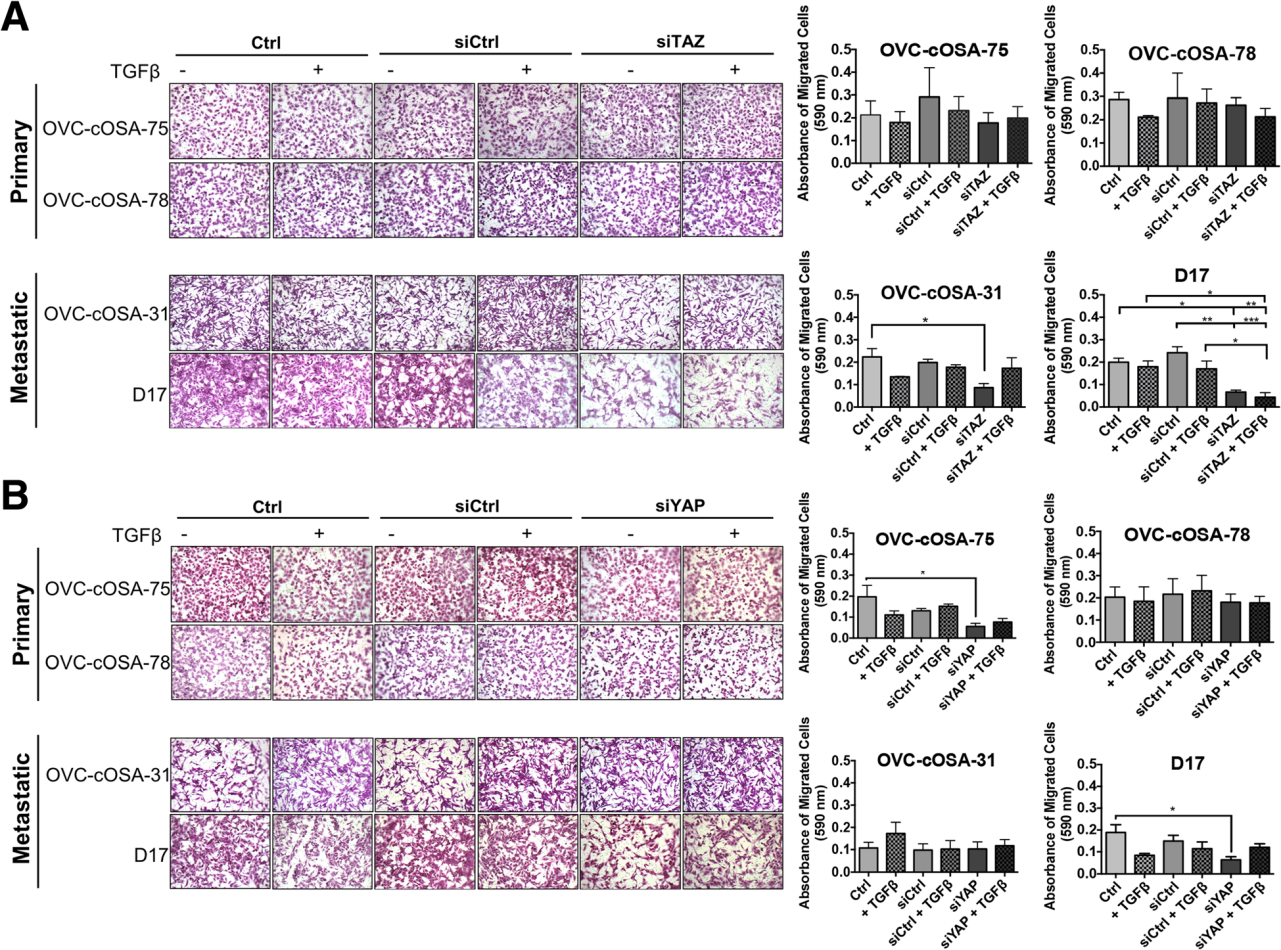
Effect of TAZ or YAP knockdown plus and minus TGFβ on transwell migration of canine OSA cell lines. Representative images of migrated cells stained with 0.1% crystal violet taken with 10X objective for primary cell lines (**a**) and metastatic cell lines (**b**). Graphs depict the absorbance values of migrated of cells, as determined by extraction of crystal violet dye from transwells using 10% acetic acid and spectrophotometer reading at 590 nm. Readings were first blank-corrected to insert containing no cells. Error bars depict average ± SEM from at least three independent experiments plated in duplicate. Asterisks depict statistical differences as determined by a two-way ANOVA with post-hoc Tukey-Kramer, * indicates *p* < 0.05, ** indicates *p* < 0.010, *** indicates *p* < 0.0010. TAZ significantly reduced migration in the OVC-cOSA-31 and D17 cell lines, while YAP knockdown reduced migration in OVC-cOSA-75 and D17 cell lines

To determine the role of TAZ, YAP, and TGFβ signalling in cell migration, the Transwell assay was performed. TAZ depletion, alone or in combination with TGFβ treatment, did not have an effect on the migratory behavior of primary tumour-derived cell lines (Fig. 9a), but significantly decreased migration in the metastatic cell lines. TAZ depletion significantly decreased cell migration in the OVC-cOSA-31 cell line when compared to the control (*p* = 0.03). However, when knockdown was combined with TGFβ in OVC-cOSA-31, cell migration increased to levels similar to those of the controls. In the D17 cell line, TAZ depletion resulted in a significant reduction in cell migration as compared to Ctrl (*p* = 0.0191) and siCtrl (*p =* 0.0024). When TAZ depletion was combined with TGFβ treatment (siTAZ + TGFβ), there was a significant decrease when compared to Ctrl + TGFβ (*p* = 0.0169), and the siCtrl group alone or in combination with TGFβ (*p =* 0.0009 and *p* = 0.0267, respectively) (Fig. 9a). These results suggest that TAZ mediates migratory properties independent of TGFβ signalling activation in D17 cells. YAP depletion did not have a noticeable effect on cell migration in the primary tumour-derived OSA cell line OVC-cOSA-78, and in the metastasis-derived OVC-cOSA-31 (Fig. 9b). However, YAP depletion significantly reduced migration in primary tumour-derived OVC-cOSA-75 (*p* = 0.0210), and metastasis-derived D17 cells (*p* = 0.0296), when compared to Ctrl (Fig. 9b). These results suggest that the role of TAZ and YAP in cell migration is cell line dependent, with predominant effects on TAZ in the metastatic cell lines that may or may not be modulated by TGFβ signalling activation.

## Discussion

Canine osteosarcoma is an aggressive, highly metastatic disease that lacks reliable prognostic factors. Due to the reported independent roles of TGFβ signalling and TAZ/YAP in promoting OSA progression, as well as their ability to crosstalk, this study aimed to investigate their cooperative role in canine OSA. To explore this possibility, we first employed a trial tissue microarray that contained 16 appendicular canine OSA patients that underwent SOC treatment. It was found that a majority of patients (approximately 60%) had high levels of pSmad2, TAZ and YAP expression. The high levels of nuclear pSmad2 observed in a high proportion of the tumours indicate active canonical signalling in canine OSA, and this is in agreement with previous reports implicating TGFβ signalling in both human [35, 36] and canine [11] OSA pathogenesis. Our choice for assessing total TAZ and YAP levels, including both nuclear and cytoplasmic protein, was based on the prominent role that proteasome-dependent degradation plays in the regulation of TAZ activity (reviewed in Yu et al. 2015 [37]). The proportion of TAZ and YAP positive tumours we observed using Allred’s scoring method, 56% and 64%, respectively, was similar to the 60% reported for total combined TAZ/YAP expression in conventional human OSA, and to the 67% reported for nuclear TAZ expression in high grade human OSA [23, 24].

There were no significant associations found between high pSmad2, TAZ or YAP levels and ALP status, nor between pSmad2, TAZ or YAP levels and histologic grade (Table 3). We recently reported that neither of the commonly used grading systems for canine OSA, nor serum ALP, are predictive for clinical outcome following SOC treatment [38]. Although we conducted the current study before the results of our grading study were complete, it is not surprising that we did not find correlations between expression levels of pSmad2, TAZ, or YAP, and grade or serum ALP. No previous studies looked for associations between TGFβ signalling status, TGFβ serum levels, TAZ or YAP and the aforementioned grading systems for canine OSA. A study in human OSA patients found a significant association between high levels of tumour TGFβ1 and high histologic grade [7]. Similarly, in human OSA, YAP1 expression has been correlated with high Enneking stages (II and III) [39].

The prognostic value of serum ALP, on the other hand, has been reiterated in recent canine osteosarcoma studies [40–42], although, as mentioned above, our recently published study found no predictive value of serum ALP in canine OSA patients receiving SOC [38]. However, the lack of associations observed between ALP status and pSmad2, TAZ, or YAP levels, is in agreement with previous reports that investigated OSA cells derived from dogs with known ALP status. Specifically, OSA cells derived from canine patients with differing serum ALP levels displayed no differences in migration, invasion and chemosensitivity when evaluated in vitro [43]. Similarly, Rodrigues and colleagues reported that serum ALP is not associated with specific gene expression patterns or intrinsic differences between patient-derived OSA cells [44]. Yet, different gene expression profiles were shown to be useful in separating canine OSA patients into short and long-term survivors [45]. These findings suggest that serum ALP levels may not provide insight on osteosarcoma cell behavior as it relates to molecular and signalling profiles. Taken together with the above-discussed controversies, our data might prove useful to future studies aimed at establishing the prognostic utility or ALP or tumour grading.

There were no significant differences in time to metastasis or overall patient survival when the markers of interest were evaluated independently. Although not statically significant, when considering the Kaplan-Meier curves in Fig. 2a, the borderline significant *p* value for pSmad2 (in relation to overall survival) and the divergent curves, suggest that patients with high levels of YAP or pSmad2 have a shorter time to metastasis and overall survival. However, these trends were not observed when considering TAZ levels alone. Interestingly, when pSmad2 and YAP were evaluated in combination (Fig. 2b, right), there was a significant difference in time to metastasis (*p* = 0.0058) and overall survival (*p* = 0.0002), with a better outcome observed in patients with lower levels of both markers. When TAZ and pSmad2 levels were considered in combination, the trends improved compared to when TAZ levels were evaluated alone, but not compared to when pSmad2 was evaluated alone. This suggests that TAZ may not have the strength that pSmad2 or YAP have as potential prognostic factors.

Of note, the potential association between YAP, metastasis and survival appears to be limited to protein levels, as we did not observe any associations with metastasis, survival or recurrence when we looked at mRNA levels of *TAZ/WWTR1* or *YAP1* alone or in combination with a TGFβ signature, using published datasets (Figs. 3 and 4, and data not shown). This is in agreement with the lack of reports of similar associations in human OSA, and possibly reflects the fact that a major determinant of whether or not TAZ and YAP protein levels are sustained to ultimately lead to a cellular response depends on post-translational modifications, such as phosphorylation events, which direct the proteasome-dependent degradation of these transcriptional co-activators. This is in turn controlled by complex signaling networks that activate or inhibit the Hippo signaling cascade, and can also be different for YAP as compared to TAZ (reviewed in [46]).

Since we recognized the limitations in interpreting the TMA findings due to the small sample size, we explored further the relationship between TAZ, YAP and TGFβ signalling in vitro using four independently derived canine OSA cell lines: two originating from a primary tumour (OVC-cOSA-75 and OVC-cOSA-78) and two derived from metastatic, secondary tumours (OVC-cOSA-31 and D17). We utilized siRNAs specific to TAZ and YAP (siTAZ, siYAP) and treated or not treated these cells with TGFβ1, to investigate the biological function of this crosstalk.

We first sought to characterize the response of cell lines to TGFβ1 treatment, and to look at basal and activated levels of canonical mediators pSmad2, Smad2, pSmad3, Smad3, and the effect of TGFβ signalling activation on TAZ and YAP levels. As expected and in agreement with previous studies [11], all cell lines expressed both receptors (Fig. 5a) and responded to TGFβ1 treatment as indicated by the increase in Smad3 and Smad2 phosphorylation (Fig. 5b and c). Also in agreement with the literature is the observed increase in TAZ protein levels in response to TGFβ1 treatment [14], while this was not seen for YAP.

We first examined the effect of TAZ and YAP knockdown in cell viability in the presence or absence of chemotherapy and in the presence of absence of active TGFβ signaling. The results showed that both TAZ and YAP modulate cell viability, but the effect of TAZ was limited to the D17 cell line (Fig. 6). As the Hippo pathway and TGFβ signalling have been documented to be important cell cycle regulators [47, 48], and to understand better whether the effects of knockdown on cell viability reflected changes in cell proliferation, we investigated the role of TGFβ-Hippo signalling on mitotic activity by examining pHH3 fluorescent labeling. Interestingly, TGFβ treatment did not have a significant effect on the percentage of mitotically active cells in the majority of cell lines, with the exception of D17. In agreement with the results when examining cell viability, TAZ knockdown significantly decreased the percentage of mitotically active cells only in the D17 cell line, where it also inhibited the growth promoting effect of TGFβ (Fig. 7a). This effect could possibly reflect the fact that exogenous TGFβ does not significantly enhance TAZ levels in D17 cells, where TAZ expression might be induced by TGFβ produced by the cells themselves (Fig. 8). The intrinsic levels of TAZ in this cell line thus appear to contribute to both, its basal proliferation and that induced by exogenous TGFβ, either by influencing canonical signal outcome (via facilitating Smad nuclear retention or transcriptional activity), or by interfering with non-canonical signaling.

YAP knockdown did not significantly reduce the percentage of mitotically active cells in any of the cell lines tested, although a slight decrease was observed in the metastatic cell lines in relation to both controls (Fig. 7b). Previous cell cycle analyses demonstrated that YAP1 knockdown decreases cell proliferation by arresting cells in the G0/G1 phase while decreasing the number of cells in the S phase [49]; however, the proportion of cells in the G2/M phase was similar when comparing the YAP knockdown and control group. These results suggest that YAP may have a more dominant role in mediating cell cycle progression (which is in line with its ability to induce expression of cyclin D1) [49], and could explain why the effects of YAP knockdown on proliferation did not reach significance in our study, as pHH3 positivity reflects the number of mitotically active cells. Taken together with the consistent reduction of viability across cell lines upon YAP knockdown (Fig. 6b), and the significance of such effects in the presence of Doxorubicin, our results suggest a key role for YAP in canine osteosarcoma survival and possibly in proliferation, and are in agreement with the associations of YAP with time to metastasis and overall survival suggested by the TMA analysis.

The results of the Transwell assay showed that TAZ knockdown significantly decreased cell migration in both of the metastasis-derived cell lines, D17 and OVC-cOSA-31 (Fig. 9a), and in D17 this was also significant in the presence of TGFβ. YAP knockdown, on the other hand, inhibited migration of two cell lines independently of their primary or secondary site of origin (Fig. 9b), and similar to what was observed for TAZ, this effect on migration was only significant for one cell line in the presence of TGFβ. Taken together with the effect of TAZ knockdown only in the viability and proliferation of D17 cells, these results suggest that TAZ effects of OSA cells were specific to metastasis-derived cell lines, and whether or not active TGFβ signaling modulates the effect that TAZ or YAP has in OSA cell migration is cell line dependent.

A role for TAZ in migration of canine OSA cells is in agreement with a previous report in human osteosarcoma, in which TAZ was found to mediate migratory and colony forming ability of OSA cells [50]. TAZ role in migration was found to be mediated, at least in part, via a positive feedback loop involving miR-135b [50]. TAZ induced the expression of miR-135b, and miR-135b overexpression was able to rescue the pro-tumourigenic functions in cells with depleted TAZ. A similar interaction was reported between miR-224 and TAZ [51]. The significant effect of YAP depletion on the migratory behavior of canine OSA cells is also in agreement with a previous report by Zhang and colleagues, who demonstrated that YAP1 depletion decreased invasiveness in human OSA [39].

Interestingly, when comparing the patient data with the results observed in vitro, the results obtained were not completely congruent. YAP and pSmad2 showed a potential prognostic value, but not TAZ. However, in vitro, TAZ appeared to have a functional role in modulating migration and cell proliferation in metastasis-derived cell lines. A possibility for the observed discrepancy could be due to the small patient sample size in the TMA. Although the median survival time for all patients considered was 292 days, similar to previous reports for canine appendicular OSA undergoing SOC (~ 300 days) [52], increasing the sample size could lead to greater power to recognize significant trends. In addition, although the TMA was an advantageous technique to determine the expression of these markers and its potential prognostic value, it only allows for the evaluation of a small portion of tumour tissue. A small tumour sample may not be able to fully recapitulate the marker expression of the tumour, and OSA is notoriously heterogeneous. Another possibility could be that the total levels (nuclear and cytoplasmic) of TAZ and YAP, and not their nuclear levels, were investigated in the tumour tissue. Given that TAZ and YAP are transcription factors, its nuclear retention is important for mediating oncogenic properties [53], and have indeed been associated with poor progression free survival in OSA [24]. Thus, determining correlations between nuclear TAZ and YAP alone or in combination with pSmad2 could yield different results.

Another possibility for the observed discrepancies could be the complexity in regulatory inputs for TAZ and YAP. All in vitro experiments were completed under serum deprivation conditions, and mechanical and environmental inputs were not considered, whereas patient tissue samples provided a realistic representation of microenvironmental effects. Furthermore, only viability, proliferation, and migration were evaluated. The function of TAZ and YAP in mediating other metastasis and drug-response associated traits, such as cellular survival and stemness, should be evaluated in future studies. Nonetheless, this pilot study demonstrated that Hippo mediators, in particular YAP, mediates canine OSA cell viability and migration independent of active TGFβ signalling, and YAP and pSmad2, alone and in combination, could potentially be used as a prognostic factor. Future studies should evaluate these molecules in larger canine patient cohorts, as well as determine the effects of TAZ and YAP knockdown and/or pharmacologic targeting of these transcription factors as well as TGFβ, on additional traits of OSA progression.

## Conclusions

To the best of our knowledge, this was the first study to investigate TGFβ-Hippo signalling crosstalk in canine OSA. Assessment of pSmad2, TAZ and YAP proteins alone or in combination in a trial TMA suggests a potential benefit of using pSmad2 and YAP combined as prognostic markers for canine OSA, with low levels of both possibly indicating better prognosis. This requires further testing in a more robust patient cohort. The in vitro results indicate that both YAP and TAZ modulate metastasis-associated properties of canine osteosarcoma, with the effects of TAZ on proliferation and migration being more specific to cell lines derived from metastases. The impact of microenvironment-derived TGFβ on TAZ/YAP-dependent cellular outcomes, such as viability, migration and proliferation is context-dependent. Additional mechanistic studies with a larger number of cell lines and combined TGFβ signalling blocking strategies are required to better understand the role of TGFβ-Hippo signalling crosstalk in canine OSA.

## Methods

### Antibodies and TGFβ

Antibodies used in this study included: YAP (#14074), β actin (#4967S), pSmad2 (#3108), and Smad2 (#5339) or Smad2/3 (#5678) for pSmad2 normalization (all from Cell Signaling Technology, New England Biolabs, Ltd., Whitby, ON, Canada). TβR1 (v-22) (#sc-398) was purchased from Santa Cruz Biotechnology Inc. (Dallas, TX, USA). TβRII (ab612143), anti-Histone H3 (phospho S10, ab5176), pSmad3 (ab52903), and Smad3 (ab28379) were purchased from Abcam. TAZ/WWTR1 antibody (HPA007415) and secondary antibody, goat anti-rabbit IgG-peroxidase (A0545), were purchased from Sigma-Aldrich (Oakville, ON, Canada). rhTGFβ1 (PHG9204) was purchased from Invitrogen, Life Technologies (Burlington, ON, Canada). LY2157299 (Cayman Chemicals) was purchased from Cedarlane (Burlington, ON, Canada). Doxorubicin (Accord Healthcare, Kirkland, QC, Canada) was obtained from the Ontario Veterinary College Pharmacy.

### TMA construction

Cases were selected from the Ontario Veterinary College Health Sciences Centre (OVC-HSC) database. Formalin fixed paraffin embedded (FFPE) tissues for the selected cases were retrieved from the Animal Health Laboratory archive. Specific areas of the FFPE tissue for each individual case were selected for coring using the guidance of haematoxylin and eosin stained histologic sections. A single 1.0 mm core was taken from each donor paraffin block and transferred into the recipient block using a Pathology Devices TMArrayer™ (Pathology Devices, Westminster, MD, USA). The recipient block contained 101 available positions that included canine tissue cores from: 31 primary appendicular OSAs with 7 matched metastatic sites, 10 axial OSAs with 2 matched metastatic sites, a selection of other tumours, and normal tissue. After the TMA was constructed, a glass slide was placed on top of the block and was placed in an oven at 55 °C for 15 min to bind the cores into the surrounding paraffin. The TMA block was left to cool before removing the slide and covering the surface of the block with a paraffin wax seal. The TMA block was cut at 4 μm, then each section was mounted on a positively charged slide and baked in an oven overnight at 37 °C.

### Antibody specificity

To determine antibody specificity, TAZ and YAP immunoblotting was completed on lysates obtained from 5 cell lines grown under standard conditions (see culture conditions in corresponding section below). These included three in-house derived cell lines (OVC-cOSA-31, OVC-cOSA-75, and OVC-cOSA-78), as well as D17 and Abrams (Additional file 1: Figure S1). The latter cell lines were a generous gift from Dr. Anthony Mutsaers (Department of Biomedical Sciences, University of Guelph), although D17 is also commercially available. For the pSmad2 immunoblotting, the same cell lines were serum starved for 6 h and either treated or not treated with 5 ng/mL TGFβ1 for 24 h prior to lysate collection.

### Immunohistochemistry of TMAs

Verification of antibody specificity, as well as optimization of antigen retrieval time and antibody concentration was first performed, according to manufacturer’s instructions, resulting in the protocol described next. First, slides were deparaffinized in 3 xylene washes (2 min each), and subsequently hydrated in 3 washes in 100% isopropanol (2 min each), and one wash in 70% isopropanol (2 min) and deionized water (2 min). Slides were then blocked in 3% hydrogen peroxide for 20 min to prevent endogenous peroxidase activity and rinsed with deionized water for 5 min. Antigen retrieval was performed with Sodium Citrate buffer pH 6.0 (Invitrogen, Burlington, ON, Canada) at 95 °C for 15 and 10 min for the pSmad2 and TAZ TMA, respectively. Following a cool-down step at room temperature for 30 min, slides were subsequently washed 3 times with Tris Buffer Saline containing 0.1% Tween (TBST), pH = 7.4 (Fisher Scientific, Ottawa, Canada) for 2 min each. To prevent antibody nonspecific binding, slides were incubated with 5% normal goat serum (Vector Laboratories, Burlington, ON, Canada) diluted in phosphate buffered saline (PBS) (Lonza, Walkersvilla, MD, USA) for one hour at room temperature in a humidified chamber. Slides were then incubated with 300 μL of primary antibody, 1:800 dilution of polyclonal rabbit phospho-Smad2 Ser465/467 (Cell Signaling, cat no. 3101), 1:200 dilution of polyclonal rabbit TAZ (Sigma-Aldrich HPA007415), or 1:100 dilution of monoclonal rabbit YAP (Cell Signaling, cat no. D8H1X) overnight at 4 °C. Slides were washed 3 times with TBST (2 min each) and incubated with biotinylated anti-rabbit IgG secondary antibody (Vector Laboratories Inc., Burlingame, CA) for one hour at room temperature. Slides were subsequently washed 3 times with TBST (2 min each) and incubated with the avidin-biotin complex (R.T.U VectaStain Kit Elite ABC Reagent, Vector Laboratories Inc., Burlingame, CA) for one hour at room temperature. To detect immunolabelling, slides were washed 3 times with TBST (2 min each) and incubated with 2,2′-diaminobenzidine (DAB) substrate, prepared with the DAB substrate kit (Vector Laboratories, Burlington ON, Canada) for 1 min. Slides were immersed in deionized water to stop the reaction, counterstained in haematoxylin (Thermo Fisher Scientific, Waltham, MA, United States) and dehydrated with 3 washes in 100% isopropanol and 3 washes in xylene (2 min each). Slides were coverslipped using Richard-Allan Scientific Cytoseal XXL mounting media (Thermo Fisher Scientific, Waltham, MA, United States).

### Tumour grading

Tumour grading was completed by a veterinary pathologist (C.R.S) using full-face tumour sections following the scoring system outlined by Kirpensteijn et al. [28] and Loukopoulos et al. [29] at 40X (2.37 mm^2^) objective lens.

### Immunohistochemistry Quantification & Statistical Analyses

The TMA was viewed and imaged with a Leica DM LM light Microscope (Leica Microsystems, Wetzlar, Germany) and each core was subsequently imaged using the QICAM digital camera and QCapture v.2.99.5 (QImaging, Surrey, Canada). Five images were taken randomly throughout each core of the TMA at 400X magnification in 8-bit format, which were used to score both nuclear Smad2 and total (nuclear plus cytoplasmic) TAZ and YAP immunoreactivity using the Allred method [54]. This method is based on two categories, the proportion score (PS) and the intensity score (IS), which are summed to the total score (TS), a maximum value of 8. The PS is based on percentage of positively stained cells 0–5 (0: 0%, 1: 0–1%, 2: 1–10%, 3: 10–33%, 4: 33–66% and 5: 67–100%) and IS ranges from 0 to 3 (0: absent, 1: weak, 2: intermediate, 3: strong). Initially, five cores (twenty-five images) were manually scored to obtain a PS, which was then compared to the PS obtained using the publicly available image processing software ImageJ v1.49 (http://imagej.nih.gov) with ImmunoRatio (Advanced Mode) plugin (http://jvsmicroscope.uta.fi). ImmunoRatio computes a “DAB/nuclear area” percentage that was then converted to a PS, as outlined by the Allred method above. As the PS determined by visual assessment and with ImageJ were found to be similar, ImageJ was used for the remainder of the quantification, while the IS was visually assessed. During this time, the observer was blinded to patient data until all quantification was performed. The TS was averaged for all fives images per core, resulting in a single score for each core. The TS was averaged for all cores on the TMA to establish the cut-off point of 6.00. All scores were compared to the established cut-off and were classified as high for pSmad2, TAZ and YAP expression if their TS were greater than or equal to 6.00, and conversely classified as low if less than 6.00 (Fig. 1a). Cores that were damaged during processing were excluded from analysis, such that 16 cases were available for TAZ and pSMAD2 and 14 were available for YAP analysis. Overall survival was defined as time from diagnosis to death, and time to metastasis was defined as the number of days from diagnosis to the confirmation (radiographic or postmortem examination) or strong suspicion (radiographic or clinical signs) of metastatic disease. Thus, dogs that died without confirmation of metastasis were censored from the analysis of time to metastasis and no dogs were assumed to have metastasis unless there was evidence for this. Dogs that died of causes unrelated to OSA were censored from overall survival analyses.

### Associations of *TAZ/WWTR1* and *YAP* mRNA expression with metastasis and survival

Processed datasets (GSE14827, GSE32981, GSE39058 and GSE27217) and corresponding gene expression and clinical annotation files were downloaded from the Gene Expression Omnibus (GEO). Data was log2 transformed if needed, and in the case of microarrays with multiple probe sets mapping to a single gene, the probe set with the highest variance across samples was selected. To test the association of *TAZ/WWTR1* or *YAP* with metastasis, expression values were compared using Welch’s t-test or ANOVA. For survival analysis, samples were split into High and Low expression groups using the median as a bifurcation point and Kaplan-Meier curves were compared using the log-rank test. All graphical and statistical analyses were performed using GraphPad Prism version 5.0 software (La Jolla, CA, USA).

### Canine OSA cell lines and culture conditions

Four different canine osteosarcoma cell lines were used for these studies: the commercially available D17 (OSA lung metastases) and the OVC-cOSA series, OVC-cOSA-31 (OSA lung metastases), OVC-cOSA-75 (primary OSA, distal tibia) and OVC-cOSA-78 (primary OSA, proximal humerus). The OVC-cOSA series of cell lines were generated at GAW Laboratory (Department of Pathobiology, University of Guelph, Guelph, Canada). Cells were maintained as monolayers in HyClone™ DMEM/High Glucose media (Sigma Aldrich), supplemented with 10% FBS (Fisher Scientific) and HyClone™ 1% Penicillin/Streptomycin (Sigma Aldrich), and cultured at 37 °C in a humidified incubator, in the presence of 95% atmospheric air and 5% CO_2_. Cells were passaged once monolayers reached 100% confluency. Monolayers were first washed with PBS (Sigma Aldrich) and then detached with 1X Trypsin-EDTA (Sigma Aldrich) diluted in PBS and neutralized with media. Cells were then re-plated into fresh media at 1:4 split ratio. Media was changed every 3–4 days.

### Cell lysate collection and immunoblotting

Cells were lysed with lysis buffer containing the following components: 1X lysis buffer (Cell Signaling Technology), 1 mM PMSF (Sigma Aldrich), 2 μg/mL aprotinin (Sigma Aldrich), 1% Phosphatase Inhibitor Cocktail (Sigma Aldrich), and 1 mM sodium orthovanadate (New England BioLabs). To collect lysates, dishes were placed on ice, washed with PBS, and lysis buffer supplemented with inhibitors was added to the plate for five minutes and subsequently harvested and incubated for thirty minutes. Lysates were centrifuged at 15000 rpm for 20 min at 4 °C. The supernatant was aliquoted and stored at − 80 °C until further use. Protein concentration was determined prior to Western Blotting using a Bradford assay (Bio-Rad, Mississauga, ON, Canada). A standard Western Blot protocol was then used to determine the levels of proteins of interest. For this purpose, 25 μg – 30 μg of protein lysates were resolved in a 10% polyacrylamide gel (Bio-Rad, Mississauga, ON, Canada) and transferred to a PVDF membrane (Sigma Aldrich) by wet transfer at 100 V for 2 h. Membranes were washed briefly with Tris Buffered Saline + 0.01% Tween (pH = 7.6, TBST) and blocked with either 5% skim milk or 5% bovine serum albumin (BSA) prepared in TBST, according to manufacturer’s recommendations, for one hour at room temperature with gentle rocking. Membranes were then incubated with primary antibody (see catalog # in first section of Methods) diluted in blocking solution at the following dilutions: TAZ (1:40,000), YAP (1:1000), β-actin (1:5000), pSmad2 (1:1000), Smad2 (1:2000), pSmad3 (1:3000), Smad3 (1:3000), TβRI (1:1000) and TβRII (1:500) overnight at 4 °C on a rocking platform. The next day, membranes were washed three times with TBST (10 min/wash) and incubated with HRP-secondary antibody for 1 h at room temperature with rocking. Membranes were washed again three times with TBST (10 min/wash) and then incubated with Luminata Forte Western HRP Substrate (Fisher Scientific) for two minutes before imaging with ChemiDoc (BioRad). Densitometry was performed with ImageLab v4.0.1 (BioRad). All bands were normalized to β-actin, or their respective native protein, in the case of phosphorylated proteins.

### siRNAs and transfection

The siRNAs used were purchased from Integrated DNA Technologies (IDT) (Coralville, IA, USA). All sequences were custom designed using the DsiRNA design tool on the IDT website. The accession number for canine TAZ and YAP mRNA sequences were first obtained using NCBI BLAST and then inputted into the DsiRNA design tool on IDT’s website. Sequences generated from the website were randomly selected and are shown in Additional file 4: Table S1. Before experimentation, oligos were resuspended in Nuclease-Free Duplex Buffer (catalogue no. 11–01–03-01, IDT) at a concentration of 5 μM according to manufacturer’s protocol. Optimization experiments were performed to determine optimal concentrations before use.

For siRNA transfection, cells were seeded at a concentration of 275, 000 cells in 35 mm dishes (for the Transwell assay) or 25,000 cells in 8 well chamber slides (for immunofluorescence). siRNA transfection was completed with Lipofectamine® 3000 (Invitrogen) according to manufacturer’s protocol using a total siRNA concentration previously optimized for each cell line. For TAZ, a total concentration of 24 nM (12 nM of Duplex 1 and 3) was used for OVC-cOSA-78, 30 nM (15 nM of Duplex 1 and 3) for OVC-cOSA-75 and D17, and 24 nM (12 nM of Duplex 1 and 2) for OVC-cOSA-31. For YAP, 30 nM (15 nM of Duplex 1 and 3) was used for all cell lines. Twenty-four hours post transfection, cells were serum-starved (0.2% FBS and 1% Pen/Strep) for 6 h and then treated with 5 ng/mL TGFβ for 24-h. Post TGFβ treatment and depending on the assay, cells were either seeded in transwells for migration analysis, or fixed for immunofluorescence-based analysis of cell proliferation via immunolabelling of phospho-Histone H3 (pHH3).

### Cell viability assay and doxorubicin treatment

Cells were transfected with siRNA as described above and then counted and seeded in a 96-well plate at varying densities, which were previously optimized: 40,000 cells/mL (D17), 80,000 cells/mL (OVC-cOSA-31) or 50,000 cells/mL (OVC-cOSA-75). After cells were allowed to adhere, Doxorubucin (kept at a stock concentration of 2 mg/mL), was directly added to the plate at the respective IC50 doses previously calculated for each cell line: 30 μM (D17), 26 μM (OVC-cOSA-31) or 60 μM (OVC-cOSA-75); media alones was added to the control (non-Doxorubucin treated) cells. Twenty-four hours after treatment, 15 μL of a working solution of resazurin (5 mg/mL diluted in PBS; obtained from Sigma catalog no. R7017) was added to each well and allowed to incubate at 37 °C for 6 h. Fluorescence was measured at 530/590 nm using a BioTek Synergy HT plate reader and Gen5 software (BioTek). Values obtained were blank-corrected to the media only control. Experimental groups were plated in technical duplicate and two independent experiments were completed.

### Transwell migration assay

Cells treated as described above were counted and seeded at 2 × 10^4^ cells/200 μL of media in Corning™ 8 μm pore inserts (Fisher Scientific) and placed into a 24-well Corning™ companion plate (Fisher Scientific) containing regular growth media (10% FBS, 1% Pen/Strep). Cells were incubated for 24 h, after which the media was removed from the insert and non-migrated cells were removed from the top of the insert with a Q-tip moistened in PBS. Migrated cells were stained with 1% crystal violet for 25 min at room temperature with gentle rocking. Inserts were then rinsed and allowed to dry overnight. The inserts were then imaged using an inverted light microscope at 4X objective lens. To quantify the degree of cell movement, crystal violet stain was extracted with 10% acetic acid diluted in de-ionized water with vigorous rocking for 15 min. The extracted dye was then read at 590 nm with a spectrophotometer. All readings were blank corrected with an insert containing no cells. All experimental groups were seeded in duplicate and extracted dye readings were performed in triplicate.

### Immunofluorescence Staining & Quantification of nuclear pHH3

Cells were fixed with 4% paraformaldehyde diluted in PBS for 15 min at room temperature and subsequently washed with PBS. Cells were then permeabilized with ice-cold methanol for 15 min, washed 3 times with PBS and blocked with 5% normal donkey serum (Sigma) for 1 h at room temperature. Cells were then incubated with pHH3 antibody diluted in 5% normal donkey serum at the following dilutions: OVC-cOSA-75 (1:5000), OVC-cOSA-78 (1:10,000), D17 and OVC-cOSA-31 (1: 20,000) overnight at 4 °C. The following day, cells were washed with PBS, and incubated with Alexa-Fluor 488 (Invitrogen) at room temperature for 1 h in the dark. Cells were then washed with PBS and incubated with 0.3 μM DAPI (Fisher) for 10 min at room temperature. Following this, cells were washed with PBS, and slides mounted with Dako fluorescent mounting medium. Cells were visualized with an epifluorescent microscope and imaged at 20X objective magnification. The total number of cells in the image was determined by counting the number of DAPI-stained nuclei in a 20X image using Nucleus Counter (ImageJ) software. The number of pHH3 positive cells was assessed visually. The number of relative pHH3 positive cells was determined by dividing the number of pHH3 positive cells by the number of nuclei present in the field of the view and multiplying by 100 to determine the percentage of mitotic cells. Five images were taken per experimental group and the average percentage was calculated.

### TGFβ receptor I inhibitor (LY2157299) experiments

D17 cells were siRNA-transfected as described above in either 35 mm dishes (for protein analysis) or 8 well chamber slides (for pHH3 staining). Twenty hours post transfection, cells were serum starved for 6 h and subjected to one of four treatments: 5 ng/mL TGFβ, 10 μM of the TGFβ receptor I inhibitor LY2157299 (LY), a combination of TGFβ and LY inhibitor, or serum starvation media (media plus 0.2% FBS) alone (control). Twenty-four hours after treatment, protein lysates were extracted, or the immunofluorescence protocol explained above was performed.

### Statistical analyses

Chi square test was used to assess the association between pSmad2, TAZ, YAP and histologic grade, metastasis and alkaline phosphatase (ALP) status using GraphPad Prism v6.0c. Kaplan-Meier plots and log-rank (Mantel-Cox) tests were used to determine the correlations of pSmad2, TAZ, and YAP levels and time to metastasis and overall survival using GraphPad Prism v6.0c. For these analyses, the number of days was calculated from the date of diagnosis with OSA (radiographic or histologic) to the endpoint of confirmation of clinical metastasis detection through radiography or clinical presentation (days to metastasis) or date of death due to OSA (overall survival). The endpoint of the study was June 12, 2018; if records did not indicate the patient experienced the event, they were censored accordingly. For in vitro studies assessing the effect of TGFβ on Smad activation, TAZ and YAP expression, significant differences in protein expression were determined using an independent *t-*test between control and TGFβ-treated cells. A two-way ANOVA and a Tukey-Kramer post hoc test were used to assess the effect of TGFβ and TAZ or YAP knockdown on cell migration and proliferation using GraphPad Prism v6.0c. For cell viability experiments, a one-way ANOVA Kruskal-Wallis test and a Dunn-Sidak post hoc was used to compare treatments to control within each, the minus Doxorubicin and the plus Doxorubicin group. All tests completed were two-sided, with a *p-*value < 0.05 considered statistically significant.

## Additional files

Additional file 1: **Figure S1.** Immunoblotting of TAZ (WWTR1) and YAP in canine osteosarcoma cell lines to determine antibody specificity. TAZ antibody (cat no. HPA007415, Sigma-Aldrich) was used at a 1:80,000 dilution. The blot demonstrated a strong band at the predicted weight of 55 kDa (arrow) and slight reactivity to YAP. YAP antibody (cat no. D8H1X, Cell Signalling), used at a 1:1,000 dilution, identified a strong band at the predicted weight of 65 kDa (arrow). (PNG 9754 kb)

Additional file 2: **Figure S2.** Representative immunoblots and densitometry demonstrating reduction in TAZ protein levels post siRNA transfection at 24 hours. TAZ levels were decreased with siRNA treatment by varying levels, as indicated by the percentages, when compared to the siRNA control (siCtrl), while YAP levels remained fairly consistent. Experimental groups were normalized to loading control β-actin. Graphs depict the average fold change in TAZ or YAP expression relative to siCtrl ± SEM from three independent experiments. (PDF 527 kb)

Additional file 3: **Figure S3.** Representative immunoblots and densitometry demonstrating reduction in YAP protein levels post siRNA transfection at 24 hours. YAP levels were decreased with siRNA treatment by varying levels, as indicated by the percentages, when compared to the siRNA control (siCtrl), while TAZ levels were not affected. Experimental groups were normalized to loading control β-actin. Graphs depict the average fold change in TAZ or YAP expression relative to siCtrl ± SEM from three independent experiments. (PDF 15825 kb)

Additional file 4: **Table S1.** Duplex Sequences. (DOCX 18 kb)

## Abbreviations

ALP: Alkaline phosphatase
OSA: Osteosarcoma
SOC: Standard of care
TAZ: Transcriptional co-activator with a PDZ binding motif
TEAD: TEA domain DNA-binding family of transcription factors
TGFβ: Transforming growth factor beta
TMA: Tissue microarray
YAP: Yes associated protein

## References

[1] Rowell JL, McCarthy DO, Alvarez CE (2011). Dog models of naturally occurring cancer. Trends Mol Med.

[2] Chun R, Kurzman ID, Couto CG, Klausner J, Henry C, MacEwen EG (2000). Cisplatin and doxorubicin combination chemotherapy for the treatment of canine osteosarcoma: a pilot study. J Vet Intern Med.

[3] McMahon M, Mathie T, Stingle N, Romansik E, Vail D, London C (2011). Adjuvant carboplatin and gemcitabine combination chemotherapy postamputation in canine appendicular osteosarcoma. J Vet Intern Med.

[4] Buijs JT, Stayrook KR, Guise TA (2011). TGF-beta in the bone microenvironment: role in breast Cancer metastases. Cancer Microenviron.

[5] Wu M, Chen G, Li YP (2016). TGF-beta and BMP signaling in osteoblast, skeletal development, and bone formation, homeostasis and disease. Bone Res.

[6] Gilbert RWD, Vickaryous MK, Viloria-Petit AM (2016). Signalling by Transforming. Growth Factor Beta Isoforms in Wound Healing and Tissue Regeneration. J Dev Biol.

[7] Franchi A, Arganini L, Baroni G, Calzolari A, Capanna R, Campanacci D, Caldora P, Masi L, Brandi ML, Zampi G (1998). Expression of transforming growth factor beta isoforms in osteosarcoma variants: association of TGF beta 1 with high-grade osteosarcomas. J Pathol.

[8] Kloen P, Gebhardt MC, Perez-Atayde A, Rosenberg AE, Springfield DS, Gold LI, Mankin HJ (1997). Expression of transforming growth factor-beta (TGF-beta) isoforms in osteosarcomas: TGF-beta3 is related to disease progression. Cancer.

[9] Chen Y, Guo Y, Yang H, Shi G, Xu G, Shi J, Yin N, Chen D (2015). TRIM66 overexpresssion contributes to osteosarcoma carcinogenesis and indicates poor survival outcome. Oncotarget.

[10] Li F, Li S, Cheng T (2014). TGF-beta1 promotes osteosarcoma cell migration and invasion through the miR-143-versican pathway. Cell Physiol Biochem.

[11] Portela RF, Fadl-Alla BA, Pondenis HC, Byrum ML, Garrett LD, Wycislo KL, Borst LB, Fan TM (2014). Pro-tumorigenic effects of transforming growth factor beta 1 in canine osteosarcoma. J Vet Intern Med.

[12] Zhang H, Wu H, Zheng J, Yu P, Xu L, Jiang P, Gao J, Wang H, Zhang Y (2013). Transforming growth factor beta1 signal is crucial for dedifferentiation of cancer cells to cancer stem cells in osteosarcoma. Stem Cells.

[13] Singh A, Settleman J (2010). EMT, cancer stem cells and drug resistance: an emerging axis of evil in the war on cancer. Oncogene.

[14] Varelas X, Sakuma R, Samavarchi-Tehrani P, Peerani R, Rao BM, Dembowy J, Yaffe MB, Zandstra PW, Wrana JL (2008). TAZ controls Smad nucleocytoplasmic shuttling and regulates human embryonic stem-cell self-renewal. Nat Cell Biol.

[15] Varelas X (2014). The hippo pathway effectors TAZ and YAP in development, homeostasis and disease. Development.

[16] Cui CB, Cooper LF, Yang X, Karsenty G, Aukhil I (2003). Transcriptional coactivation of bone-specific transcription factor Cbfa1 by TAZ. Mol Cell Biol.

[17] Hong JH, Yaffe MB (2006). TAZ: a beta-catenin-like molecule that regulates mesenchymal stem cell differentiation. Cell Cycle.

[18] Zhao L, Jiang S, Hantash BM (2010). Transforming growth factor beta1 induces osteogenic differentiation of murine bone marrow stromal cells. Tissue Eng Part A.

[19] Hiemer SE, Varelas X (2013). Stem cell regulation by the hippo pathway. Biochim Biophys Acta.

[20] Bartucci M, Dattilo R, Moriconi C, Pagliuca A, Mottolese M, Federici G, Benedetto AD, Todaro M, Stassi G, Sperati F (2015). TAZ is required for metastatic activity and chemoresistance of breast cancer stem cells. Oncogene.

[21] Cordenonsi M, Zanconato F, Azzolin L, Forcato M, Rosato A, Frasson C, Inui M, Montagner M, Parenti AR, Poletti A (2011). The hippo transducer TAZ confers cancer stem cell-related traits on breast cancer cells. Cell.

[22] Hiemer SE, Szymaniak AD, Varelas X (2014). The transcriptional regulators TAZ and YAP direct transforming growth factor beta-induced tumorigenic phenotypes in breast cancer cells. J Biol Chem.

[23] Fullenkamp CA, Hall SL, Jaber OI, Pakalniskis BL, Savage EC, Savage JM, Ofori-Amanfo GK, Lambertz AM, Ivins SD, Stipp CS (2016). TAZ and YAP are frequently activated oncoproteins in sarcomas. Oncotarget.

[24] Bouvier C, Macagno N, Nguyen Q, Loundou A, Jiguet-Jiglaire C, Gentet JC, Jouve JL, Rochwerger A, Mattei JC, Bouvard D (2016). Prognostic value of the hippo pathway transcriptional coactivators YAP/TAZ and beta1-integrin in conventional osteosarcoma. Oncotarget.

[25] Wang DY, Wu YN, Huang JQ, Wang W, Xu M, Jia JP, Han G, Mao BB, Bi WZ (2016). Hippo/YAP signaling pathway is involved in osteosarcoma chemoresistance. Chin J Cancer.

[26] Beffagna G, Sacchetto R, Cavicchioli L, Sammarco A, Mainenti M, Ferro S, Trez D, Zulpo M, Michieletto S, Cecchinato A (2016). A preliminary investigation of the role of the transcription co-activators YAP/TAZ of the hippo signalling pathway in canine and feline mammary tumours. Vet J.

[27] Guillemette S, Rico C, Godin P, Boerboom D, Paquet M (2017). In vitro validation of the hippo pathway as a pharmacological target for canine mammary gland tumors. J Mammary Gland Biol Neoplasia.

[28] Kirpensteijn J, Kik M, Rutteman GR, Teske E (2002). Prognostic significance of a new histologic grading system for canine osteosarcoma. Vet Pathol.

[29] Loukopoulos P, Robinson WF (2007). Clinicopathological relevance of tumour grading in canine osteosarcoma. J Comp Pathol.

[30] Kobayashi E, Masuda M, Nakayama R, Ichikawa H, Satow R, Shitashige M, Honda K, Yamaguchi U, Shoji A, Tochigi N (2010). Reduced argininosuccinate synthetase is a predictive biomarker for the development of pulmonary metastasis in patients with osteosarcoma. Mol Cancer Ther.

[31] Namlos HM, Kresse SH, Muller CR, Henriksen J, Holdhus R, Saeter G, Bruland OS, Bjerkehagen B, Steen VM, Myklebost O (2012). Global gene expression profiling of human osteosarcomas reveals metastasis-associated chemokine pattern. Sarcoma.

[32] Kelly AD, Haibe-Kains B, Janeway KA, Hill KE, Howe E, Goldsmith J, Kurek K, Perez-Atayde AR, Francoeur N, Fan JB (2013). MicroRNA paraffin-based studies in osteosarcoma reveal reproducible independent prognostic profiles at 14q32. Genome Med.

[33] Scott MC, Sarver AL, Gavin KJ, Thayanithy V, Getzy DM, Newman RA, Cutter GR, Lindblad-Toh K, Kisseberth WC, Hunter LE (2011). Molecular subtypes of osteosarcoma identified by reducing tumor heterogeneity through an interspecies comparative approach. Bone.

[34] Baglio SR, Lagerweij T, Perez-Lanzon M, Ho XD, Leveille N, Melo SA, Cleton-Jansen AM, Jordanova ES, Roncuzzi L, Greco M (2017). Blocking tumor-educated MSC paracrine activity halts osteosarcoma progression. Clin Cancer Res.

[35] Lamora A, Talbot J, Bougras G, Amiaud J, Leduc M, Chesneau J, Taurelle J, Stresing V, Le Deley MC, Heymann MF (2014). Overexpression of smad7 blocks primary tumor growth and lung metastasis development in osteosarcoma. Clin Cancer Res.

[36] Yang R, Piperdi S, Zhang Y, Zhu Z, Neophytou N, Hoang BH, Mason G, Geller D, Dorfman H, Meyers PA (2016). Transcriptional profiling identifies the signaling axes of IGF and transforming growth factor-b as involved in the pathogenesis of osteosarcoma. Clin Orthop Relat Res.

[37] Yu FX, Zhao B, Guan KL (2015). Hippo pathway in organ size control, tissue homeostasis, and Cancer. Cell.

[38] Schott CR, Tatiersky LJ, Foster RA, Wood GA (2018). Histologic grade does not predict outcome in dogs with appendicular osteosarcoma receiving the standard of care. Vet Pathol.

[39] Zhang YH, Li B, Shen L, Shen Y, Chen XD (2013). The role and clinical significance of YES-associated protein 1 in human osteosarcoma. Int J Immunopathol Pharmacol.

[40] Boerman I, Selvarajah GT, Nielen M, Kirpensteijn J (2012). Prognostic factors in canine appendicular osteosarcoma - a meta-analysis. BMC Vet Res.

[41] Garzotto CK, Berg J, Hoffmann WE, Rand WM (2000). Prognostic significance of serum alkaline phosphatase activity in canine appendicular osteosarcoma. J Vet Intern Med.

[42] Schmidt AF, Nielen M, Klungel OH, Hoes AW, de Boer A, Groenwold RH, Kirpensteijn J, Investigators VSSO (2013). Prognostic factors of early metastasis and mortality in dogs with appendicular osteosarcoma after receiving surgery: an individual patient data meta-analysis. Prev Vet Med.

[43] Holmes KE, Thompson V, Piskun CM, Kohnken RA, Huelsmeyer MK, Fan TM, Stein TJ (2015). Canine osteosarcoma cell lines from patients with differing serum alkaline phosphatase concentrations display no behavioural differences in vitro. Vet Comp Oncol.

[44] Rodrigues LC, Holmes KE, Thompson V, Piskun CM, Lana SE, Newton MA, Stein TJ (2016). Osteosarcoma tissues and cell lines from patients with differing serum alkaline phosphatase concentrations display minimal differences in gene expression patterns. Vet Comp Oncol.

[45] Selvarajah GT, Kirpensteijn J, van Wolferen ME, Rao NA, Fieten H, Mol JA (2009). Gene expression profiling of canine osteosarcoma reveals genes associated with short and long survival times. Mol Cancer.

[46] He M, Zhou Z, Shah AA, Hong Y, Chen Q, Wan Y (2016). New insights into posttranslational modifications of hippo pathway in carcinogenesis and therapeutics. Cell Div.

[47] Ehmer U, Sage J (2016). Control of proliferation and Cancer growth by the hippo signaling pathway. Mol Cancer Res.

[48] Zhang Y, Alexander PB, Wang XF. TGF-beta family signaling in the control of cell proliferation and survival. Cold Spring Harb Perspect Biol. 2017;9(4). 10.1101/cshperspect.a022145 PMC5378054 27920038

[49] Yang Z, Zhang M, Xu K, Liu L, Hou WK, Cai YZ, Xu P, Yao JF (2014). Knockdown of YAP1 inhibits the proliferation of osteosarcoma cells in vitro and in vivo. Oncol Rep.

[50] Shen S, Huang K, Wu Y, Ma Y, Wang J, Qin F, Ma J (2017). A miR-135b-TAZ positive feedback loop promotes epithelial-mesenchymal transition (EMT) and tumorigenesis in osteosarcoma. Cancer Lett.

[51] Ma J, Huang K, Ma Y, Zhou M, Fan S (2017). The TAZ-miR-224-SMAD4 axis promotes tumorigenesis in osteosarcoma. Cell Death Dis.

[52] Selmic LE, Burton JH, Thamm DH, Withrow SJ, Lana SE (2014). Comparison of carboplatin and doxorubicin-based chemotherapy protocols in 470 dogs after amputation for treatment of appendicular osteosarcoma. J Vet Intern Med.

[53] Chan SW, Lim CJ, Loo LS, Chong YF, Huang C, Hong W (2009). TEADs mediate nuclear retention of TAZ to promote oncogenic transformation. J Biol Chem.

[54] Allred DC, Harvey JM, Berardo M, Clark GM (1998). Prognostic and predictive factors in breast cancer by immunohistochemical analysis. Mod Pathol.

