# Supplementary figures and images for: An evaluation of TAZ and YAP crosstalk with TGFβ signalling in canine osteosarcoma suggests involvement of hippo signalling in disease progression
Source: BMC Vet Res. 2018 Nov 26;14:365. doi: 10.1186/s12917-018-1651-5 (PMC6258471; doi:10.1186/s12917-018-1651-5)

### OVC-cOSA-75

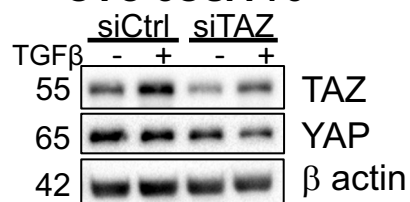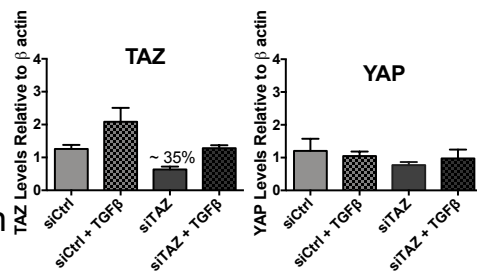

### OVC-cOSA-78

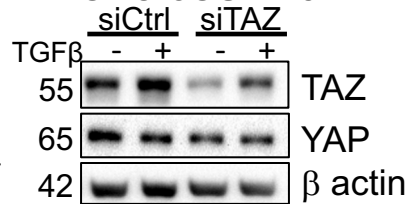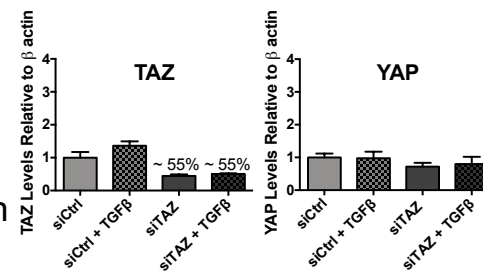

### OVC-cOSA-31

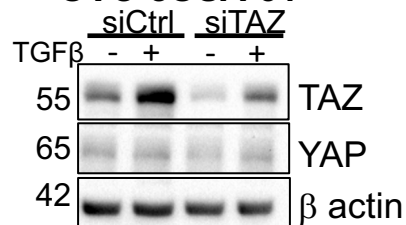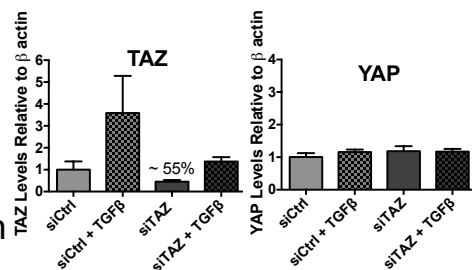

### D17

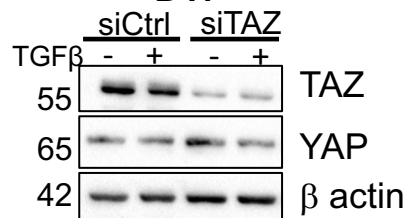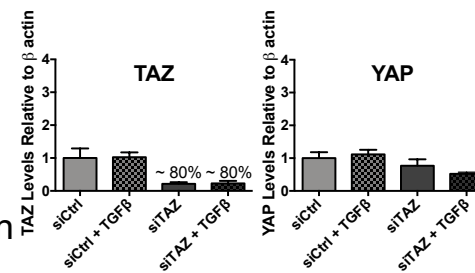

Supplement: Supplementary file 2 — Figure S2. Representative immunoblots and densitometry demonstrating reduction in TAZ protein levels post siRNA transfection at 24 hours. TAZ levels were decreased with siRNA treatment by varying levels, as indicated by the percentages, when compared to the siRNA control (siCtrl), while YAP levels remained fairly consistent. Experimental groups were normalized to loading control β-actin. Graphs depict the average fold change in TAZ or YAP expression relative to siCtrl ± SEM from three independent experiments. (PDF 527 kb) [file 12917_2018_1651_MOESM2_ESM.pdf]

### OVC-cOSA-75

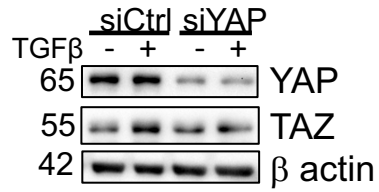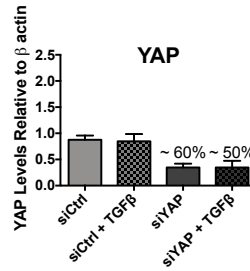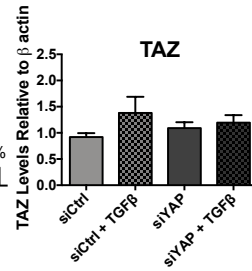

### OVC-cOSA-78

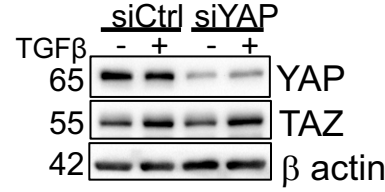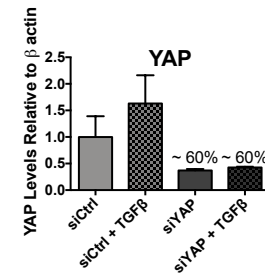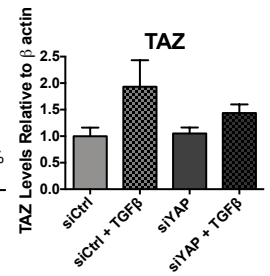

### OVC-cOSA-31

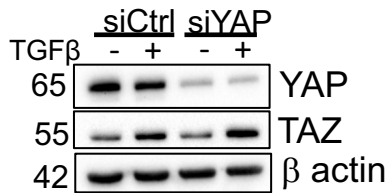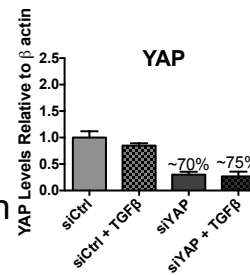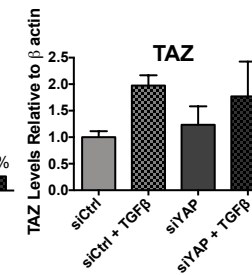

### D17

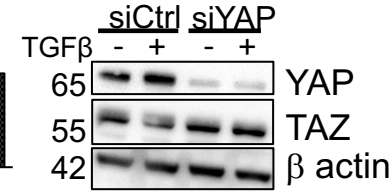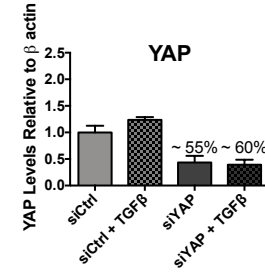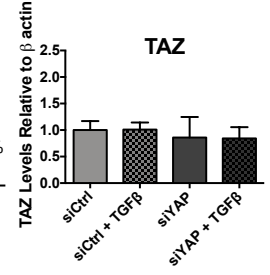

Supplement: Supplementary file 3 — Figure S3. Representative immunoblots and densitometry demonstrating reduction in YAP protein levels post siRNA transfection at 24 hours. YAP levels were decreased with siRNA treatment by varying levels, as indicated by the percentages, when compared to the siRNA control (siCtrl), while TAZ levels were not affected. Experimental groups were normalized to loading control β-actin. Graphs depict the average fold change in TAZ or YAP expression relative to siCtrl ± SEM from three independent experiments. (PDF 15825 kb) [file 12917_2018_1651_MOESM3_ESM.pdf]
