# Supplementary material for: An evaluation of TAZ and YAP crosstalk with TGFβ signalling in canine osteosarcoma suggests involvement of hippo signalling in disease progression
Source: BMC Vet Res. 2018 Nov 26;14:365. doi: 10.1186/s12917-018-1651-5 (PMC6258471; doi:10.1186/s12917-018-1651-5)
Supplement: Supplementary file 4 — Table S1. Duplex Sequences. (DOCX 18 kb) [file 12917_2018_1651_MOESM4_ESM.docx]

**Additional file 1 for:**

**An evaluation of TAZ and YAP crosstalk with TGFβ signaling in canine osteosarcoma suggests involvement of Hippo signalling in disease progression**

Anita K. Luu, Courtney R. Schott, Robert Jones, Andrew C. Poon, Brandon Golding, Roa’a Hamed, Benjamin Deheshi, Anthony Mutsaers, Geofrey A. Wood, Alicia M. Viloria-Petit.

Additional file 4**:** Table S1: **Duplex Sequences**

| TAZ Duplex 1 | Sense | rGrGrArArCrArArArUrGrUrUrGrArCrCrUrArGrGrArArCTT |
| --- | --- | --- |
|  | Antisense | rArArGrUrUrCrCrUrArGrGrUrCrArArCrArUrUrUrGrUrUrCrCrCrG |
| TAZ Duplex 2 | Sense | rCrCrArGrGrArArArUrGrUrArCrArCrGrArArArUrUrArGAC |
|  | Antisense | rGrUrCrUrArArUrUrUrCrGrUrGrUrArCrArUrUrUrCrCrUrGrGrArC |
| TAZ Duplex 3 | Sense | rGrGrArUrCrArGrArArUrGrCrGrUrCrArArGrArGrGrArGCT |
|  | Antisense | rArGrCrUrCrCrUrCrUrUrGrArCrGrCrArUrUrCrUrGrArUrCrCrGrC |
| YAP Duplex 1 | Sense | rUrArCrUrUrUrUrCrArUrGrArUrArCrArGrArArArArCrArArCrUrG |
|  | Antisense | rGrUrUrGrUrUrUrUrCrUrGrUrArUrCrArUrGrArArArArGTA |
| YAP Duplex 2 | Sense | rUrGrUrGrArUrUrCrArArGrArArGrUrArUrCrUrCrUrGrArCrCrArG |
|  | Antisense | rGrGrUrCrArGrArGrArUrArCrUrUrCrUrUrGrArArUrCrACA |
| Negative Control* | Sense | rCrGrUrUrArArUrCrGrCrGrUrArUrArArUrArCrGrCrGrUAT |
|  | Antisense | rArUrAr rGrCrGrUrArUrUrArUrArCrGrCrGrArUrUrArArCrGrArC |

*Catalog number for negative control DsiRNA: 51-01-14-04 (IDT)

**Supplemental Figure Legends**

Additional file 1: **Figure S1.** Immunoblotting of TAZ (WWTR1) and YAP in canine osteosarcoma cell lines to determine antibody specificity. TAZ antibody (cat no. HPA007415, Sigma-Aldrich) was used at a 1:80,000 dilution. The blot demonstrated a strong band at the predicted weight of 55 kDa (arrow) and slight reactivity to YAP. YAP antibody (cat no. D8H1X, Cell Signalling), used at a 1:1,000 dilution, identified a strong band at the predicted weight of 65 kDa (arrow).

Additional file 2: **Figure S2.** Representative immunoblots and densitometry demonstrating reduction in TAZ protein levels post siRNA transfection at 24 hours. TAZ levels were decreased with siRNA treatment by varying levels, as indicated by the percentages, when compared to the siRNA control (siCtrl), while YAP levels remained fairly consistent. Experimental groups were normalized to loading control β-actin. Graphs depict the average fold change in TAZ or YAP expression relative to siCtrl ± SEM from three independent experiments.

Additional file 3: **Figure S3.** Representative immunoblots and densitometry demonstrating reduction in YAP protein levels post siRNA transfection at 24 hours. YAP levels were decreased with siRNA treatment by varying levels, as indicated by the percentages, when compared to the siRNA control (siCtrl), while TAZ levels were not affected. Experimental groups were normalized to loading control β-actin. Graphs depict the average fold change in TAZ or YAP expression relative to siCtrl ± SEM from three independent experiments.
